# Dynamic electron radiation belt at Mercury controlled by solar wind driving

**DOI:** 10.1038/s41550-026-02925-3

**Published:** 2026-07-27

**Authors:** Jiutong Zhao, Ryan M. Dewey, Weijie Sun, James A. Slavin, Paul S. Szabo, Lucas Liuzzo, Nicolas André, Mathias Rojo

**Affiliations:** 1 Space Sciences Laboratory, University of California, Berkeley, Berkeley, CA USA; 2 Department of Climate and Space Sciences and Engineering, University of Michigan, Ann Arbor, MI USA; 3 Institut de Recherche en Astrophysique et Planétologie (IRAP), CNES-CNRS-Université de Toulouse, Toulouse, France; 4 Institut Supérieur de l’Aéronautique et de l’Espace (ISAE-SUPAERO), Université de Toulouse, Toulouse, France

**Keywords:** Inner planets, Space physics, Space physics

## Abstract

Electron radiation belts, reservoirs of stably trapped energetic electrons hazardous to space technology, are a prevalent feature of most magnetized planetary environments. However, their existence in the compact magnetosphere of Mercury has been a subject of debate for decades. The presence of energetic electrons was first identified by Mariner 10’s observations in the 1970s but was subsequently questioned by MESSENGER’s limited energetic particle measurements 40 years later. Here we integrate MESSENGER’s more sensitive indirect energetic electron measurements with new analysis techniques, particle simulations and theory to discover a structured radiation belt with a variable morphology subject to solar wind activity. This radiation belt can persist for several Earth days and, thus, seems to be quasi-permanent under weak solar wind forcing. However, it becomes unstable under strong driving as electron drift orbits bifurcate about the magnetic equator within the dayside magnetosphere and particles are rapidly lost. Our results establish that even compact magnetospheres can host radiation belts and, furthermore, that Mercury is a natural laboratory for radiation-belt physics usually inaccessible at Earth: one of an extreme, rapidly driven state.

## Abstract (web summary)

Mercury is found to host a radiation belt under weak solar wind driving that becomes unstable during strong forcing, providing insights into energetic particle dynamics in an extreme planetary environment.

## Main

One of the defining early discoveries of the Space Age was the existence of Earth’s radiation belts by Explorer 1 in 1958^1^. Since then, such populations of high-energy, magnetically trapped charged particles have been identified about every magnetized planet in the Solar System except for Mercury^2–5^. Mercury, whose global magnetic field was an unexpected discovery by Mariner 10 in 1974^6^, boasts the Solar System’s most compact and rapidly dynamic planetary magnetosphere driven by the intense solar wind close to the Sun^7–9^. Such solar wind forcing can, at times, produce responses resembling those of Earth during extreme Carrington-class events. Given the substantial hazards that radiation belts pose to humans and space technology, Mercury’s extreme system offers an important and unique natural space weather laboratory for studying energetic particle dynamics, particularly trapping and loss^10,11^.

Mariner 10 recorded bursts of energetic electrons in Mercury’s magnetotail analogous to Earth’s substorm injections^12,13^. Decades later, dedicated measurements from MESSENGER confirmed that the magnetosphere accelerates electrons to >100 keV (refs. ^14,15^). However, whether Mercury’s weak intrinsic dipole field can trap energetic particles has been under debate since Mariner 10’s fly-bys^5^. Indeed, more sensitive but indirect MESSENGER Neutron Spectrometer (NS) observations indicated that energetic electrons can be found at all local times and are continuously present for several minutes about the planet^16^. Nevertheless, at the nominal 20-s resolution of NS, it is difficult to distinguish between stably trapped electrons that execute several complete drift orbits and the repeated injection of rapidly lost particles. This 20-s resolution is comparable with both the ~20-s drift period of 100-keV electrons near Mercury^17^ and the ~10–20-s spacing between magnetotail injections that themselves last ~1–10 s (ref. ^18^). Higher resolution (10-ms) Gamma-Ray Spectrometer (GRS) measurements confirmed that some energetic electrons drift completely about the planet several times before being lost^17^. These GRS measurements are only partially and discontinuously available, so even though they can resolve detailed energetic electron dynamics, they are limited in evaluating the occurrence of trapped electrons and the upstream conditions that favour them.

We reconcile these indirect observations from NS and GRS to identify instances of stable energetic electron structures within Mercury’s magnetosphere and contextualize them with particle tracing simulations. We demonstrate that Mercury can host stable energetic electron populations for magnetospherically prolonged intervals (much longer than Mercury’s 2–3-min substorm cycle known as the Dungey cycle^7,19^) akin to radiation belts found throughout the Solar System. This population tends to be stable when Mercury is at aphelion under weak upstream forcing but turns unstable under extreme driving (for example, perihelion) as the electron drift orbits bifurcate.

## Results

**Fig. 1:**
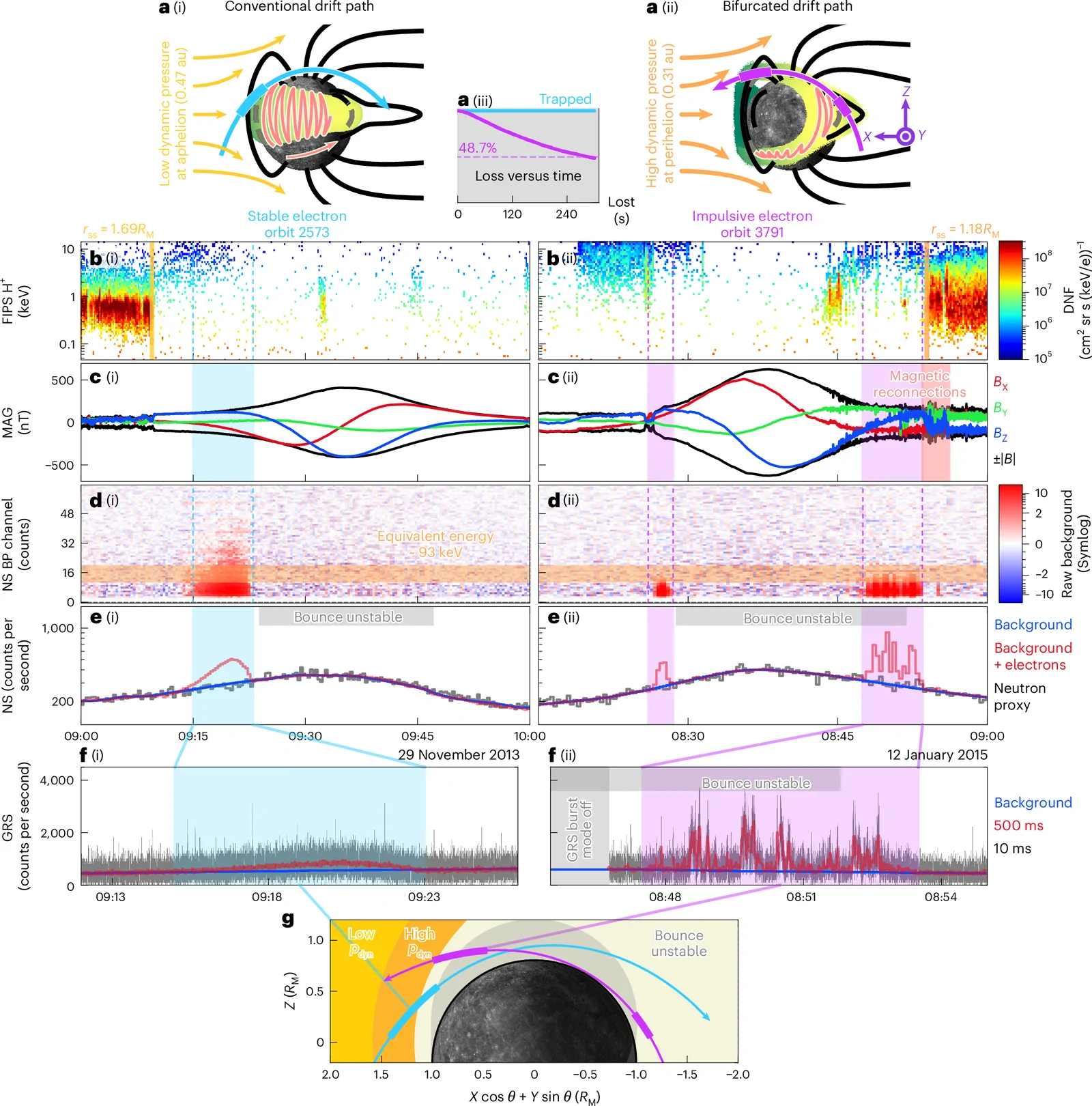
Magnetospheric energetic electrons observed by MESSENGER. **a**–**f**, Two representative events showing stable (left, 29 November 2013) and impulsive (right, 12 January 2015) electron enhancements. **a**, (i), (ii), Schematic diagrams of the two morphologies of Mercury’s electron drift shell (illustrated by green and yellow shading) from test-particle simulations. Spacecraft trajectories are overlaid for the two events. Two typical electron trajectories are shown as red solid lines. The input parameters for the test-particle simulations are provided in Extended Data Table 1. (iii) Time evolution of the surviving fraction of test particles for the two drift-shell morphologies, corresponding to the conventional drift path near aphelion and the bifurcated drift path near perihelion (heliocentric distance *r*_hel_ ≈ 0.47 au and 0.31 au, respectively). **b**, Differential proton flux measured by the Fast-Imaging Plasma Spectrometer. High-energy protons (~10 keV), resembling ring-current populations^23^, are collocated with the energetic electrons. **c**, Magnetic field in aMSM coordinates. **d**, Background-subtracted time-channel spectrogram from the NS BP detector. Higher BP channels correspond to higher electron energies. The shaded regions mark the approximate channels of 93-keV electron energy. **e**, NS count rates: blue (algorithm-defined background), red (background + electron signal) and grey (scaled proxy for planetary neutrons). The bounce unstable regions are where bouncing electrons ought to be lost in the southern hemisphere. **f**, GRS count rates: blue (background), black (background + electron signal, high resolution, 10 ms) and red (background + electron signal, 500 ms moving average). **g**, MESSENGER’s trajectory in the orbital plane. The spacecraft orbit was closely aligned with the noon–midnight plane during these orbits. Mercury USGS/NASA MESSENGER MDIS basemap data in **a** and **g** from refs. ^64–66^. DNF, differential number flux; Symlog, symmetric logarithmic scale; FIPS, Fast-Imaging Plasma Spectrometer; MAG, Magnetometer; aMSM, aberrated Mercury solar magnetospheric coordinate system.

**Extended Data Fig. 1:**
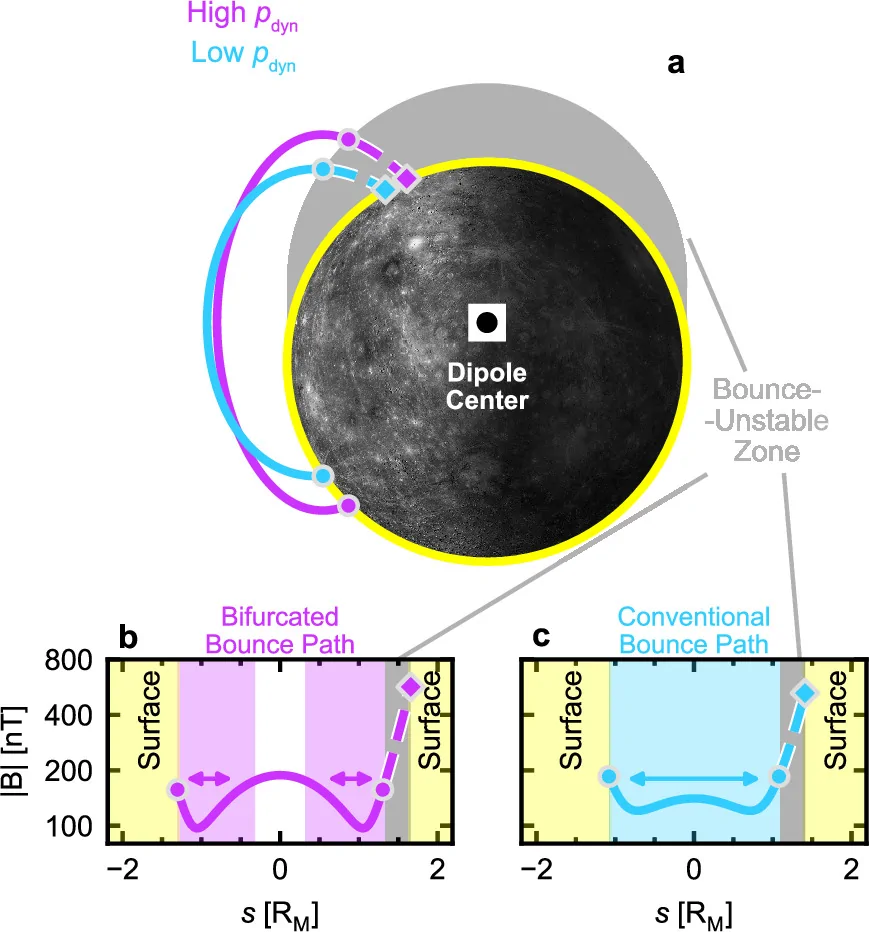
Schematic of the magnetic field line and intensity profile modeled by Mercury’s magnetospheric model. (**a**) Two modeled magnetic field lines under strong (purple) and weak (cyan) solar wind dynamic pressure conditions. Gray shading marks the bounce-unstable zone. (**b**-**c**) Magnetic field intensity along the magnetic field line, centered at the magnetic equator. Mercury USGS/NASA MESSENGER MDIS basemap data in **a** from refs. ^64–66^.

Figure 1 compares energetic electron observations during two of MESSENGER’s low-altitude magnetosphere passes. The first (left-hand column) was during an inflated magnetosphere with little variability in the kilo-electronvolt protons (Fig. 1b(i) and magnetic field (Fig. 1c(i)). A long-duration energetic electron event reaching >100 keV occurred close to the planet (Fig. 1d)(i) and Fig. 1f(i))^16,17^. The high latitude edge of this electron population closely aligns with the bounce unstable zone (‘Bounce unstable zone’ in Methods and Extended Data Fig. 1 for a definition and schematic), where the bouncing electrons ought to be lost in the southern hemisphere due to its weaker magnetic field. A signal analysis of the high-resolution GRS observations found no appreciable variation at short timescales. Test-particle tracing confirmed that these electrons were probably stably, adiabatically trapped in conventional drift orbits about the planet (Fig. 1a(i)) with negligible loss (Fig. 1a(iii)). These signatures indicate the presence of a stable energetic electron structure that probably persisted after MESSENGER left the region.

By contrast, the second event (right-hand column) was during a compressed magnetosphere with substantial reconnection-related dayside fluctuations^20^ (Fig. 1b)(ii) and Fig. 1c(ii)). NS likewise recorded a long continuous dayside event but confined to lower energy channels and with greater record-to-record fluctuations. The high-resolution GRS measurements revealed that this interval was composed of several impulsive events (Fig. 1f)(ii) with timing consistent with fresh, repeated magnetotail injections^18^. Particle tracing indicates that these electrons drifted in bifurcated or ‘Shabansky’ orbits (Fig. 1a)(ii)) with high loss rates^21–23^ (Fig. 1a(iii))). We consider that these electrons were magnetically bouncing between the magnetic equator and the northern surface and, thus, were not completely regulated by the large loss cone at the southern surface. Although these electrons completed the nightside-to-dayside portion of their drift, the frequent return of the GRS rate to background levels indicates that these electrons were not stably trapped. The high expected loss from particle tracing and the pronounced temporal variability mark these electrons as impulsive or ephemeral rather than constituting a stable, spatially organized structure.

**Extended Data Fig. 2:**
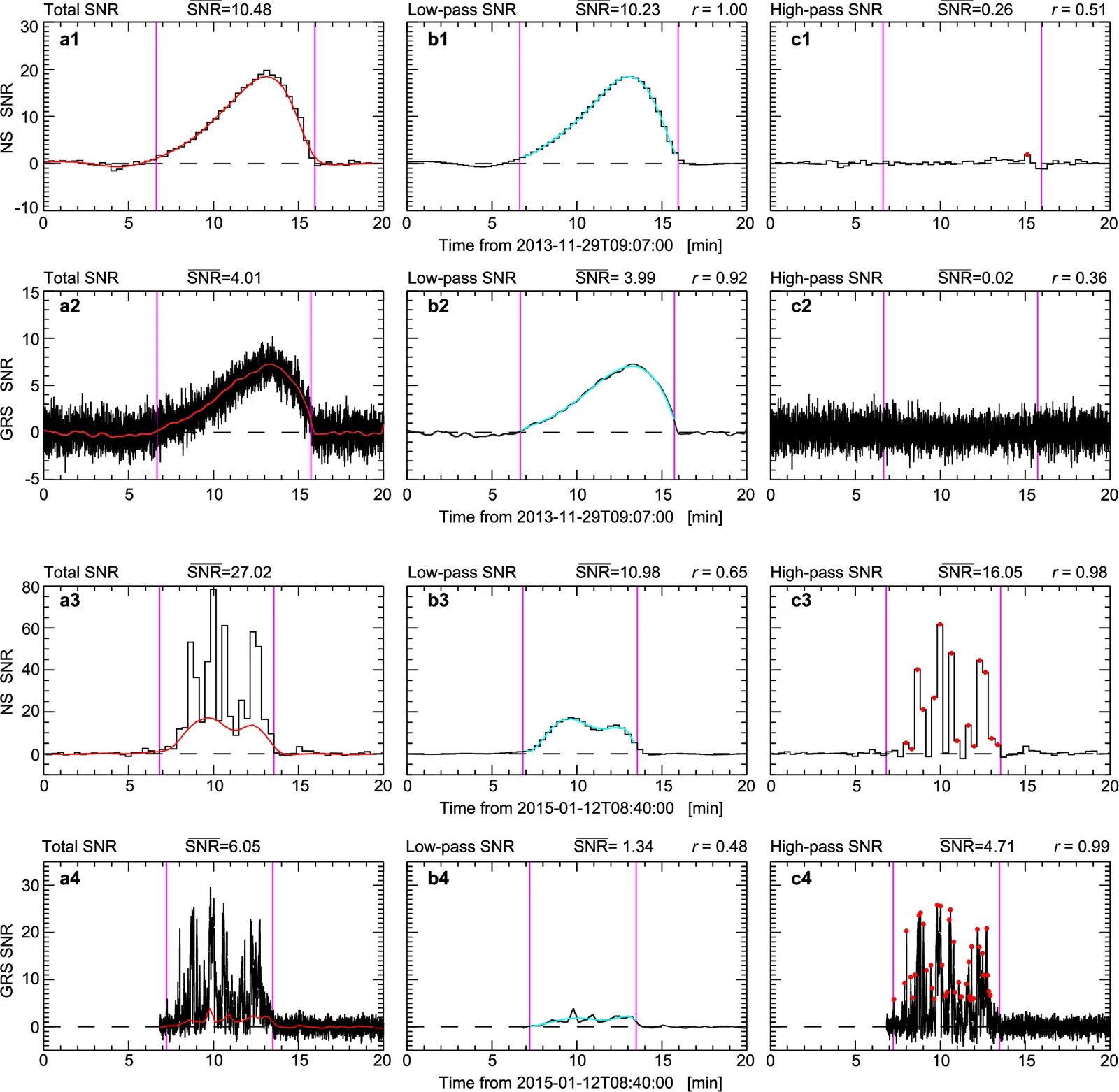
Bandpass filtering isolates short-scale variability for identifying stable energetic electron structures. **a1**-**a4**) SNR from the detection algorithms with the low-pass signal (red) overlaid. (**b1**-**b4**) Low-pass signal (black) with a high-order polynomial fit (cyan). (**c1**-**c4**) High-pass signal (black) with statistically significant peaks (red dots). Magenta lines mark the start and stop of the event or event group. The mean SNR ($\overline{{\rm{SNR}}}$) and the Pearson correlation coefficient with the total SNR (r) are provided above each panel.

**Extended Data Fig. 3:**
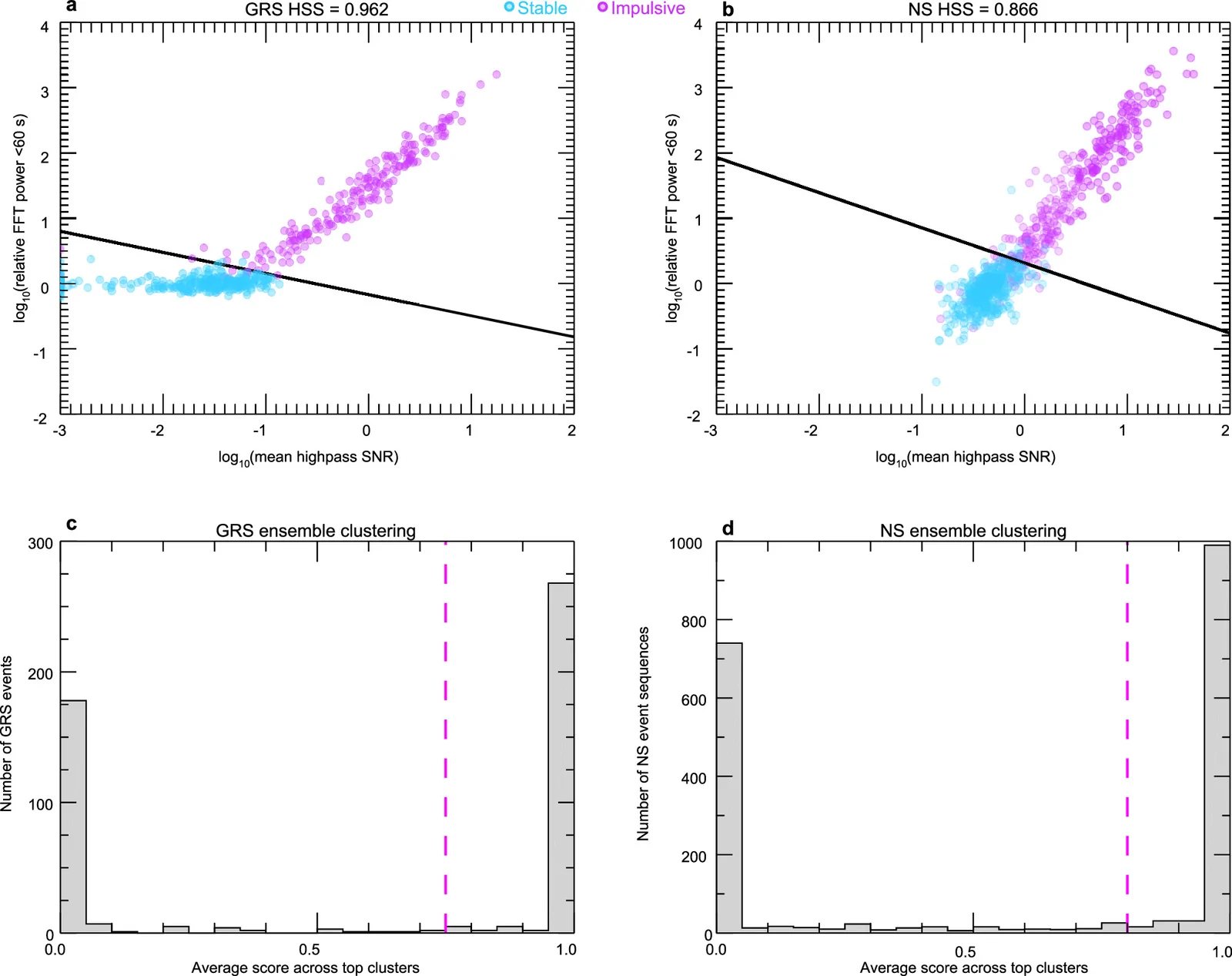
Ensemble clustering classifies GRS and NS stable energetic electron events. (**a**-**b**) Example clusters separating features between stable and impulsive events. (**c**-**d**) Distribution of average scores across the top-performing GRS and NS clusters. The vertical dashed magenta line marks the threshold for classifying stable events.

We have developed standardized detection algorithms that consistently identify intervals with energetic electrons (‘events’) and apply them to the entire MESSENGER orbital mission. As high-resolution GRS measurements are not continuously available, we used the overlapping NS and GRS event sets and clustering techniques to classify NS events as ‘stable’ or ‘impulsive’ based on signal morphology following the examples shown in Fig. 1. Details of the classification criteria and the resulting categories are provided in Methods (‘Classification of GRS and NS energetic electron events’) and Extended Data Figs. 2 and 3. Some impulsive events may correspond to long-lasting magnetosphere structures with superposed fluctuations (for example, injections overlaid on stable structures or local dynamics modifying count rates), but these instances cannot be rigorously distinguished from successive injections in these indirect data, particularly at the lower 20-s resolution.

**Fig. 2:**
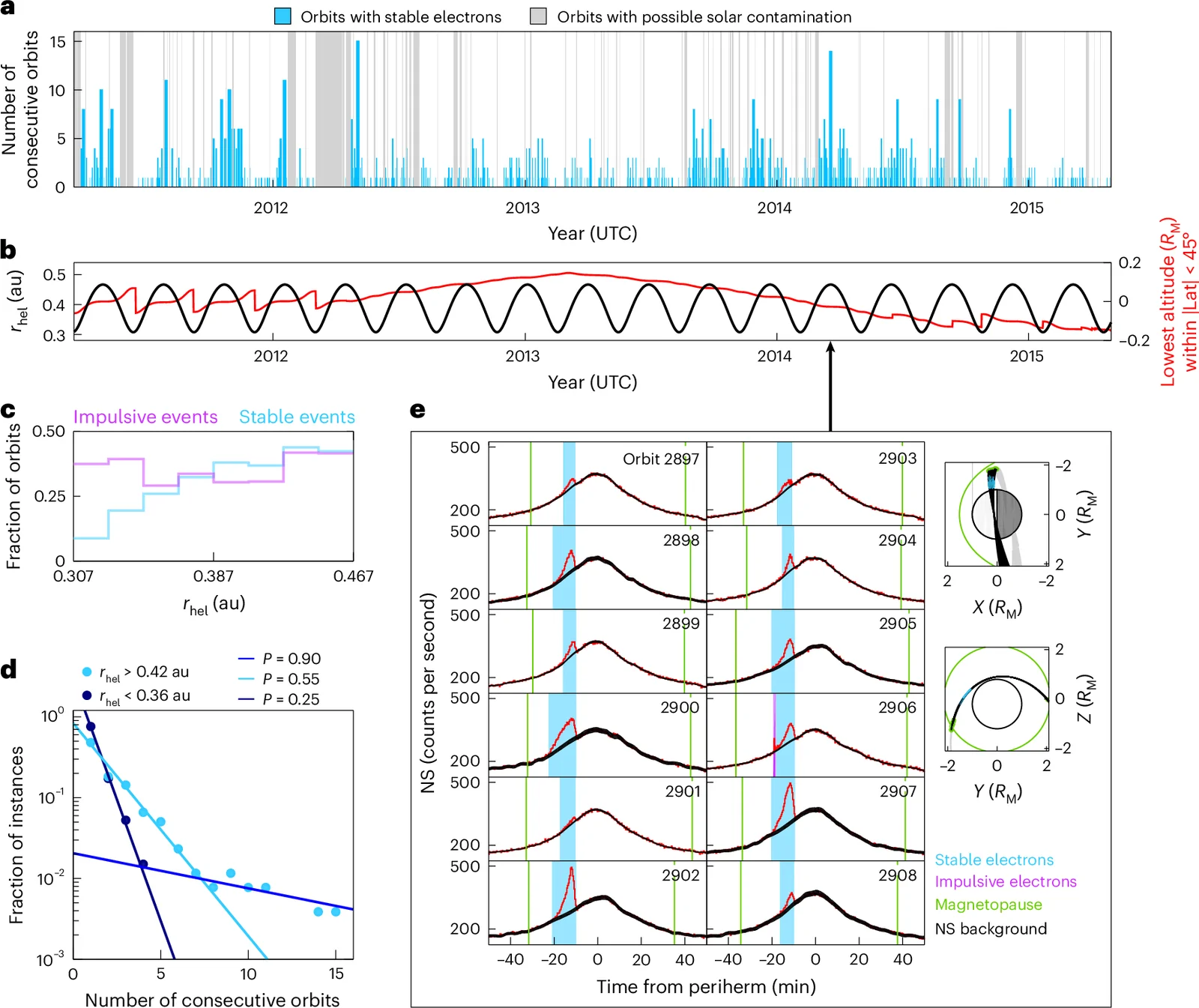
Frequency and longevity of stable energetic electron populations. **a**, Time sequence of the number of consecutive orbits that contain stable energetic electron populations (cyan bars) throughout MESSENGER’s orbital mission. Orbits contaminated by energetic solar particles or elevated suprathermal solar wind populations are excluded (grey). **b**, Mercury’s heliocentric distance from the Sun (*r*_hel_; black) and the lowest altitude sampling by the spacecraft within low latitudes (∣Lat∣ < 45°; red) over the same time interval as **a**. **c**, Fraction of orbits with stable (cyan) and impulsive (magenta) electron events as functions of *r*_hel_. **d**, Distribution of consecutive orbits with stable populations for different *r*_hel_ bins (points), with statistical model fits assuming statistically independent orbits (lines). Here *P* is the derived probability of observing a radiation belt during an individual MESSENGER magnetospheric pass by assuming statistically independent passes (‘Radiation-belt probability model’ in Methods). **e**, Sequence of 12 consecutive orbits with stable populations (orbits 2897–2908), showing NS count rates and the spacecraft trajectory relative to the magnetopause. Lat, latitude.

Figure 2a is the timeline of the stable events, which demonstrates that MESSENGER frequently encountered such structures. Mid-mission, changes in the low-latitude sampling by the spacecraft biased it against crossing these populations. Despite changes in spacecraft sampling, a clear seasonal trend is present: stable electron orbits occurred more frequently and were more probable over consecutive orbits near aphelion (Fig. 2a–c). The number of consecutive orbits follows a power-law distribution near its lower end (Fig. 2d), indicating an independent, random occurrence of the radiation belt in each orbit. However, a secondary population at aphelion of more than eight sequential orbits requires an occurrence rate of 90%, implying at least a near-continuous population during these times. One such example is shown in Fig. 2e, for which MESSENGER encountered stable electrons consistently in the low-altitude equatorial dawnside region over 12 consecutive orbits. This 96-h interval reflects a quasi-permanent radiation belt: it corresponds to ~2,000–3,000 characteristic substorm timescales, equivalent to 5–8 months at Earth. Orbit-by-orbit changes in the radiation-belt signal indicate that the structure evolved, and like radiation belts around other planets, these are probably maintained by a balance between continuous particle supply and continuing loss processes.

**Fig. 3:**
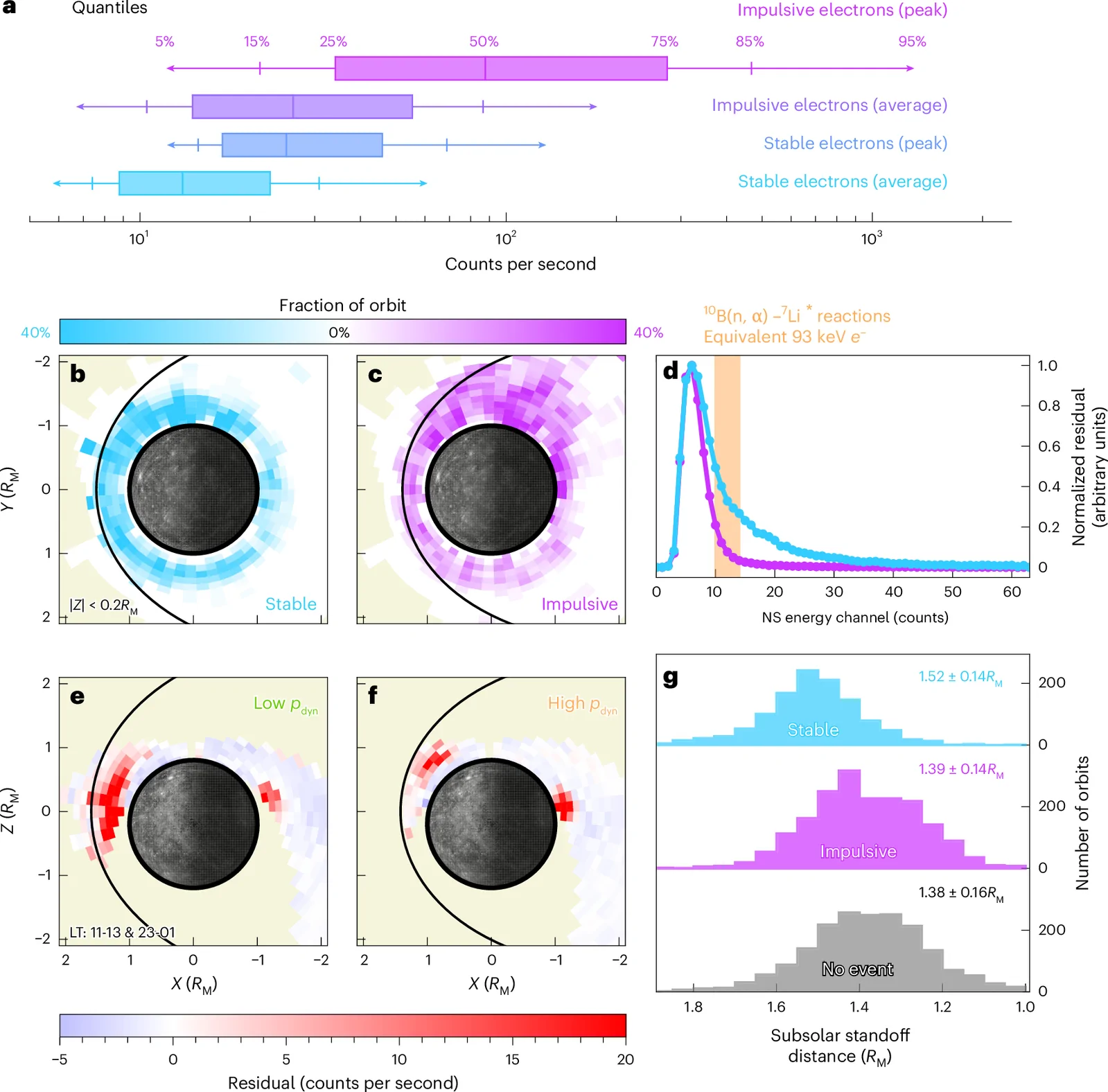
Dynamical variability, spatial occurrence, maximum energy and favourable solar wind conditions of the radiation belt. **a**, Percentile distributions of the peaks and averages for event-wise NS electron counts for impulsive and stable electron events. **b**,**c**, Spatial distributions of stable (**b**) and impulsive (**c**) electron events near the magnetic equatorial plane. **d**, Normalized residual counts contributed by each NS BP energy channel for stable and impulsive electron events. Higher BP channels correspond to higher electron energies. **e**,**f**, Spatial distributions of NS electron counts for low (**e**) and high (**f**) solar wind dynamic pressure *p*_dyn_ (with magnetopause subsolar standoff distance *r*_ss_ greater than 1.49*R*_M_ and less than 1.35*R*_M_, respectively) in the *X*–*Z* plane, where *X* is approximated by ${\rm{sgn}}(X)\sqrt{{X}^{2}+{Y}^{2}}$ to include off-meridian data. In **e** and **f**, the accumulated data are restricted to local time (LT) ranges of 23–01 h and 11–13 h. **g**, Histograms of the magnetopause subsolar standoff distances, a proxy for upstream dynamic pressure, during spacecraft orbits with stable, impulsive and no electron events. Mercury USGS/NASA MESSENGER MDIS basemap data in **b**,**c**,**e**,**f** from refs. ^64–66^.

To better understand the origin, dynamics and nature of these electron populations, we present in Fig. 3 their statistical characteristics. Stable electron orbits are clustered within the inner, dipolar region of the magnetosphere to form a ring-like distribution (Fig. 3b). Although impulsive electrons can drift around the planet, they occur predominantly in the dawnside magnetotail (Fig. 3c) where injections from substorm dipolarization events are frequent^18,24,25^. Count rates of stable electron events are systematically lower than those of impulsive events (Fig. 3a) but reach higher energies with substantial components >100 keV (Fig. 3d), implying these stable electrons are unlikely to result from direct acceleration by a single substorm dipolarization event or by the convection electric field alone. Instead, there must be a continuous energization mechanism that acts on the stably trapped electrons that impulsive electrons are lost too quickly to experience. For example, wave–particle interactions, particularly through whistler modes^26,27^, are reported to accelerate the stably trapped radiation-belt electrons up to megavolt energies in the more intense planetary fields of Earth, Jupiter and Saturn^4,28–30^, and these may be a candidate route for accelerating these electrons at Mercury.

**Fig. 4:**
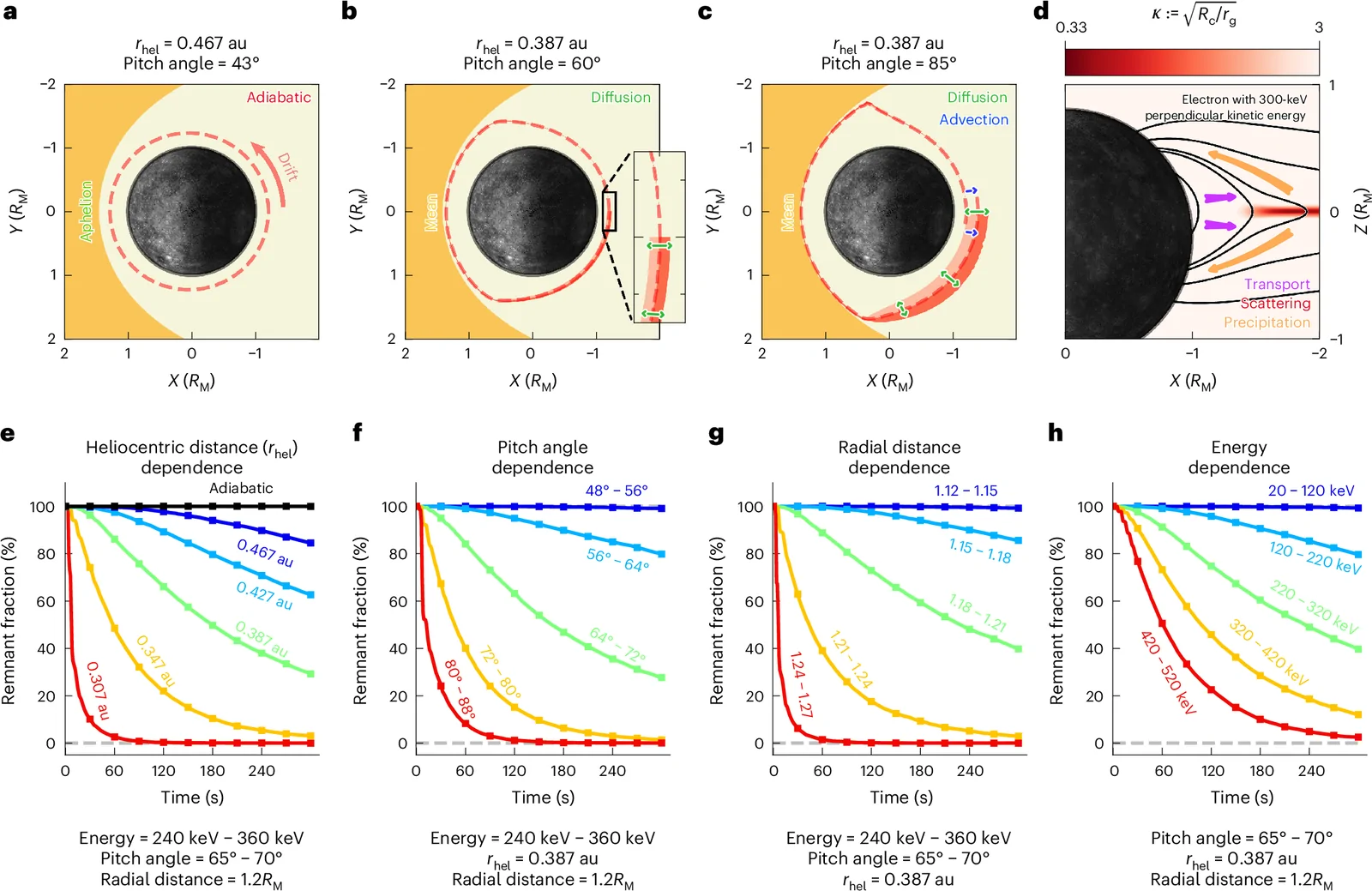
Dynamical behaviour of the radiation-belt electrons illustrated by test-particle simulations. **a**–**c**, Equatorial distributions of particle footprints after one drift cycle for three representative cases: adiabatic (**a**), diffusion-dominated (**b**) and advection-dominated (**c**). In **b** and **c**, the red-shaded regions represent the statistical distribution of test-particle equatorial footprints; in the dusk and pre-midnight sectors (enlarged in the inset of **b**), their inner edge, central line and outer edge correspond, respectively, to the 5th, 50th and 95th percentiles of the trapped electrons’ radial distance from Mercury. **d**, Distribution of 300-keV electrons *κ*, which is defined as the square root of the ratio of local magnetic curvature radius *R*_c_ and electron gyroradius *r*_g_. Electrons are considered to be lost, that is, precipitated onto nightside surface, when their *κ* < 3. **e**–**h**, Remnant fraction of radiation-belt electrons over time and its dependence on Mercury’s heliocentric distance *r*_hel_ (**e**), the equatorial pitch angle of the electrons relative to the magnetic field (**f**), radial distance near the magnetic equator (**g**) and electron kinetic energy (**h**). Input parameters for the test-particle simulations are listed in Extended Data Table 1. Mercury USGS/NASA MESSENGER MDIS basemap data in **a**–**d** from refs. ^64–66^.

**Extended Data Fig. 4:**
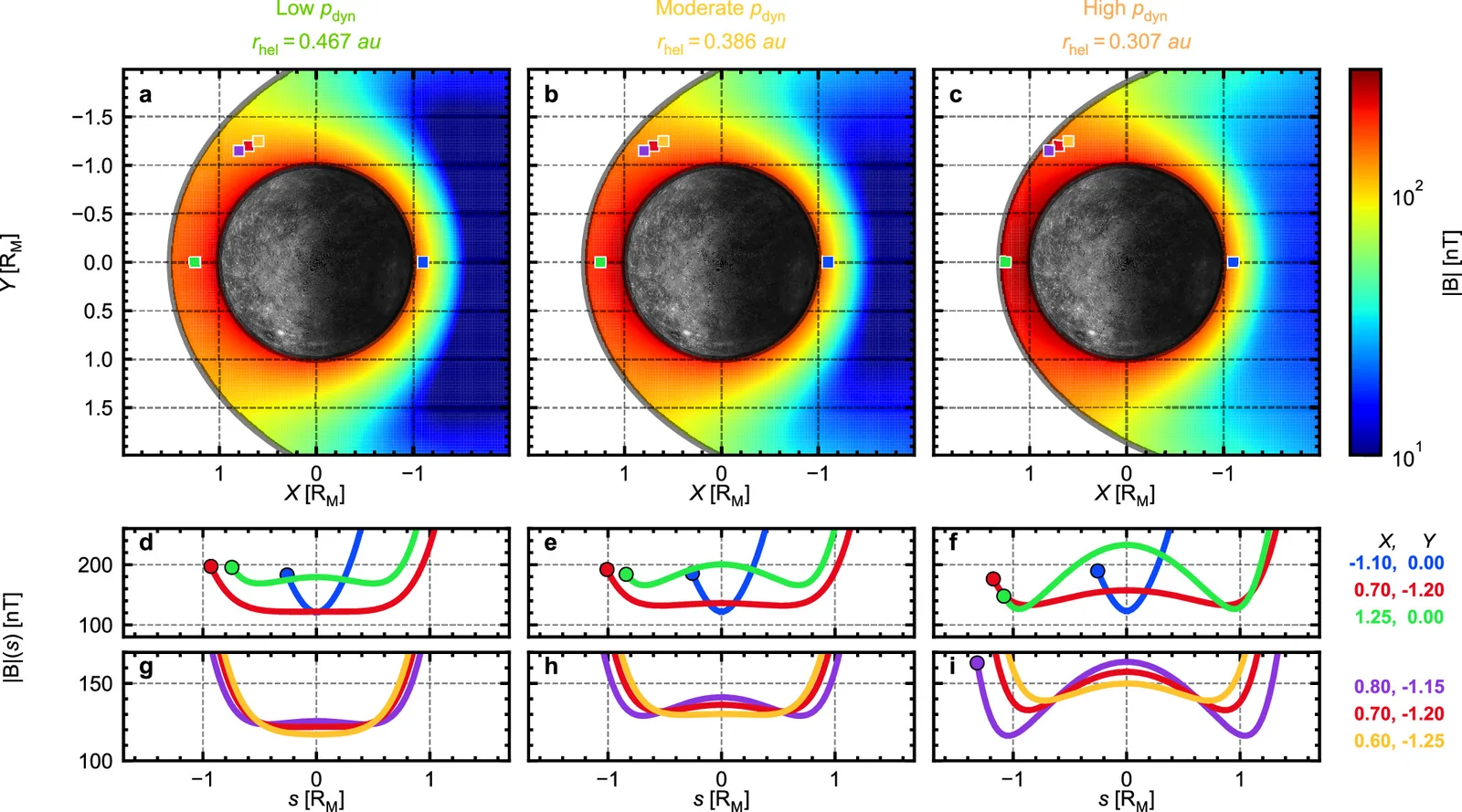
The KT17 model magnetic field distributions and configuration under different solar wind dynamic pressures. (**a**-**c**) Distributions of the magnetic field intensity at the magnetic equatorial plane. (**d**-**f**) Magnetic field intensity profiles along field lines on the dayside (green), nightside (blue), and near the bifurcation (i.e., transition) point (red). (**g**-**i**) Magnetic field intensity for three magnetic field lines around the bifurcation point. Mercury USGS/NASA MESSENGER MDIS basemap data in **a**–**c** from refs. ^64–66^.

**Extended Data Fig. 5:**
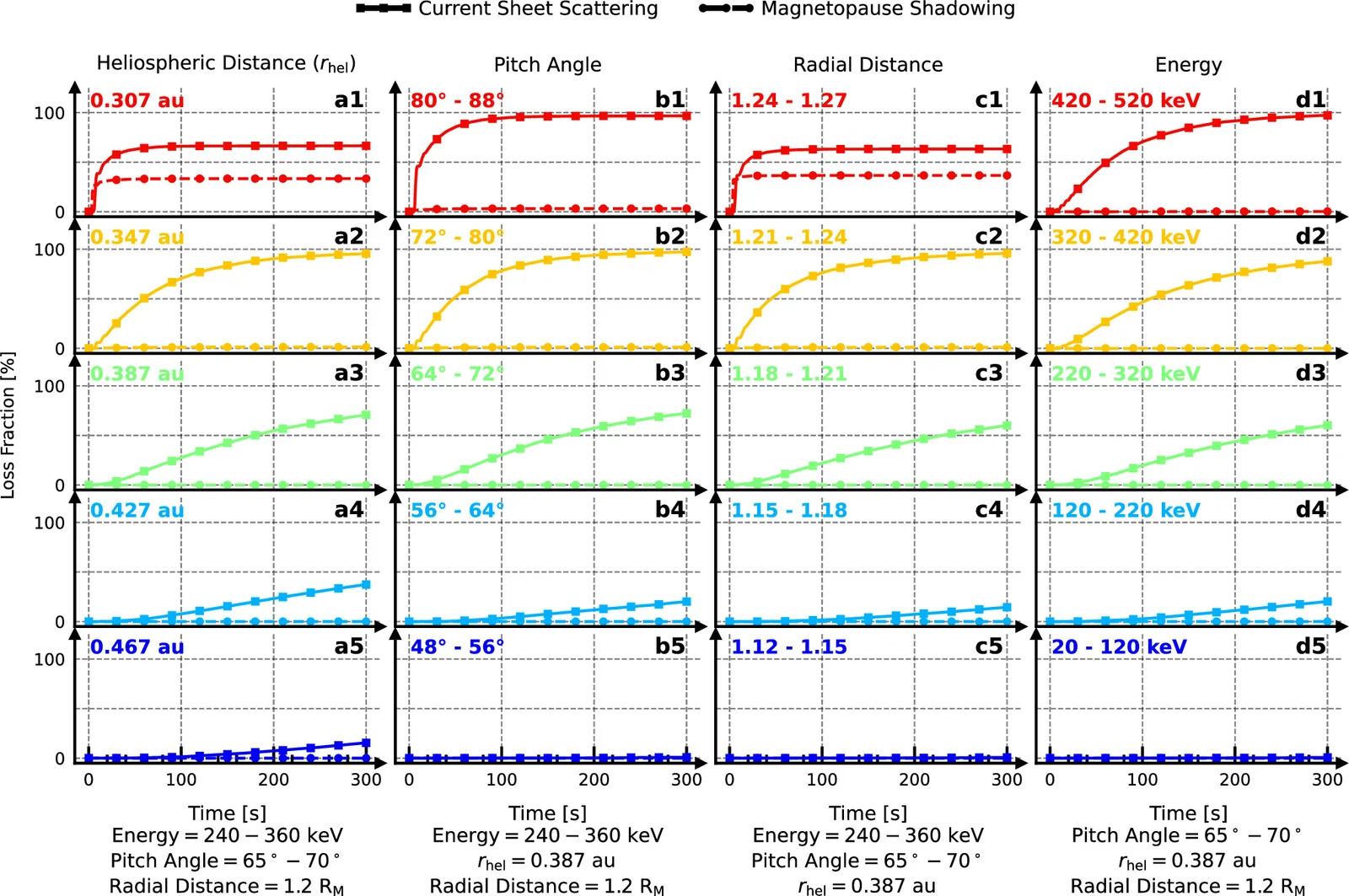
Particle loss from current sheet scattering and magnetopause shadowing under varying initial conditions. The four columns illustrate the sensitivity of particle loss to varying initial conditions: (**a1**-**a5**) heliocentric distance; (**b1**-**b5**) pitch angle; (**c1**-**c5**) radial distance; and (**d1**-**d5**) energy. For each column, the non-varying parameters are held constant, as indicated at the bottom of the respective panels. These parameter combinations correspond to those in Figs. 4e-h.

The bifurcation loss at Mercury may be organized by magnetosphere forcing; the dynamic pressure of the solar wind *p*_dyn_ seems to be deterministic for electron event type (Fig. 3g) as well as drift-orbit morphology (Fig. 3e,f) and is consistent with the seasonality discussed above. To evaluate how electron loss relates to drift-orbit morphology and depends on the solar wind dynamic pressure *p*_dyn_, we performed a parameter study using test-particle simulations (see Extended Data Table 1 for the input parameters) in a global magnetic field model (‘KT17 magnetic field model’ in Methods and Extended Data Fig. 4) of Mercury’s magnetosphere (Fig. 4). Under the weak *p*_dyn_ that is more common at aphelion^11^, a subset of electrons follow adiabatic drift orbits with negligible radial transport (Fig. 4a and Fig. 1a(i)). In the absence of rapid dynamics, these trajectories remain stably trapped over the long term (Fig. 4e). Under moderate to high *p*_dyn_, most electrons experience drift-orbit bifurcation with (inter-) intra-hemispheric bounce motion in the (nightside) dayside magnetosphere. The changing bounce geometry disrupts the bounce adiabatic invariant and alters the radial distance of the drift shell^31,32^ (‘Drift orbit bifurcation theory’ in Supplementary Information and Supplementary Fig. 2). Specifically, particles with higher energies or those located closer to the magnetopause (outer drift shell or higher *p*_dyn_) experience this orbital geometry transition more rapidly. Although particles with relatively small pitch angles (far from 90°) primarily undergo radial diffusion (Fig. 4b), those with larger pitch angles (close to 90°) experience not only enhanced radial diffusion but also radial advection (Fig. 4c). Consequently, these effects together can rapidly transport electrons either to the last closed drift shell, leading to magnetopause shadowing, or onto stretched magnetotail field lines, where the local magnetic curvature radius *R*_c_ becomes comparable with the electron gyroradius *r*_g_ ($\kappa := \sqrt{{R}_{c}/{r}_{g}} < 3$), and scattering results in their precipitation (Fig. 4d; Extended Data Fig. 5 shows the relative contributions of current-sheet scattering and magnetopause shadowing to the total loss).

Thus, loss rates depend on magnetosphere size as well as electron pitch angle, location and energy (Fig. 4e–h). A higher *p*_dyn_, larger pitch angle, distances further from the planet and higher energy all promote more rapid loss due to this non-adiabatic, bifurcated drift motion. These simulation results are consistent with energetic electron locations during low and high *p*_dyn_ (Fig. 3e,f), the conditions favourable for stable versus impulsive populations (Fig. 3g) and the seasonality of Mercury’s radiation belt (Fig. 2). Considering the complex and dynamic nature of Mercury’s magnetosphere, electrons probably experience amplified non-adiabaticity due to magnetospheric dawn–dusk asymmetry^33,34^, the crustal magnetic field^35^ and other loss mechanisms, such as scattering from wave–particle interactions^36,37^. However, the loss from drift bifurcation seems to be sufficient to explain the failed build-up of injections into a stable radiation-belt structure during periods of greater solar wind forcing.

## Conclusion

Taken together, these MESSENGER observations, statistical modelling and test-particle simulations reveal that stably drifting energetic electron populations are common and that they form a ring-like radiation belt about Mercury, even in a sompact magnetosphere. Statistical modelling and direct inference indicate that the radiation belt is a common occurrence (exists ~50% of the time near aphelion) with lifetimes typically less than 8–12 h but reaching several Earth days exceptionally. The impermanence of this structure is driven by loss rather than a lack of supply. The impulsive populations and test-particle simulations highlight the acute loss of these electrons due to drift-orbit bifurcation, which is the result of increased solar wind forcing. Due to the high ellipticity of the planetary orbit and the resulting systematic variation of *p*_dyn_, Mercury’s radiation belt exhibits a strong seasonal dependence, with a higher occurrence and stability near aphelion.

As Mercury’s radiation-belt electrons have lifetimes comparable with the injection timescales, the environment is persistently driven far from local thermodynamic equilibrium, particularly near perihelion where solar wind-driven orbit bifurcation amplifies the losses. This fundamental characteristic of Mercury’s radiation belt is qualitatively different from other radiation belts in the Solar System, as weak thermodynamic equilibrium is maintained by gradual energization, transport and loss. At Earth, wave–particle interactions play a central role in these processes, whereas at Jupiter and Saturn, cosmic ray albedo neutron decay, radial transport, interchange instabilities, corotation-driven dynamics and strong coupling to moons and rings are often dominant, with wave–particle interactions acting as one of several contributing mechanisms^4,26,27,38^. Therefore, Mercury serves as a natural test bed for investigating radiation-belt physics under extreme external driving that is rare at other planets, and the results can be applied to magnetized planets beyond the Solar System^39^.

**Extended Data Fig. 6:**
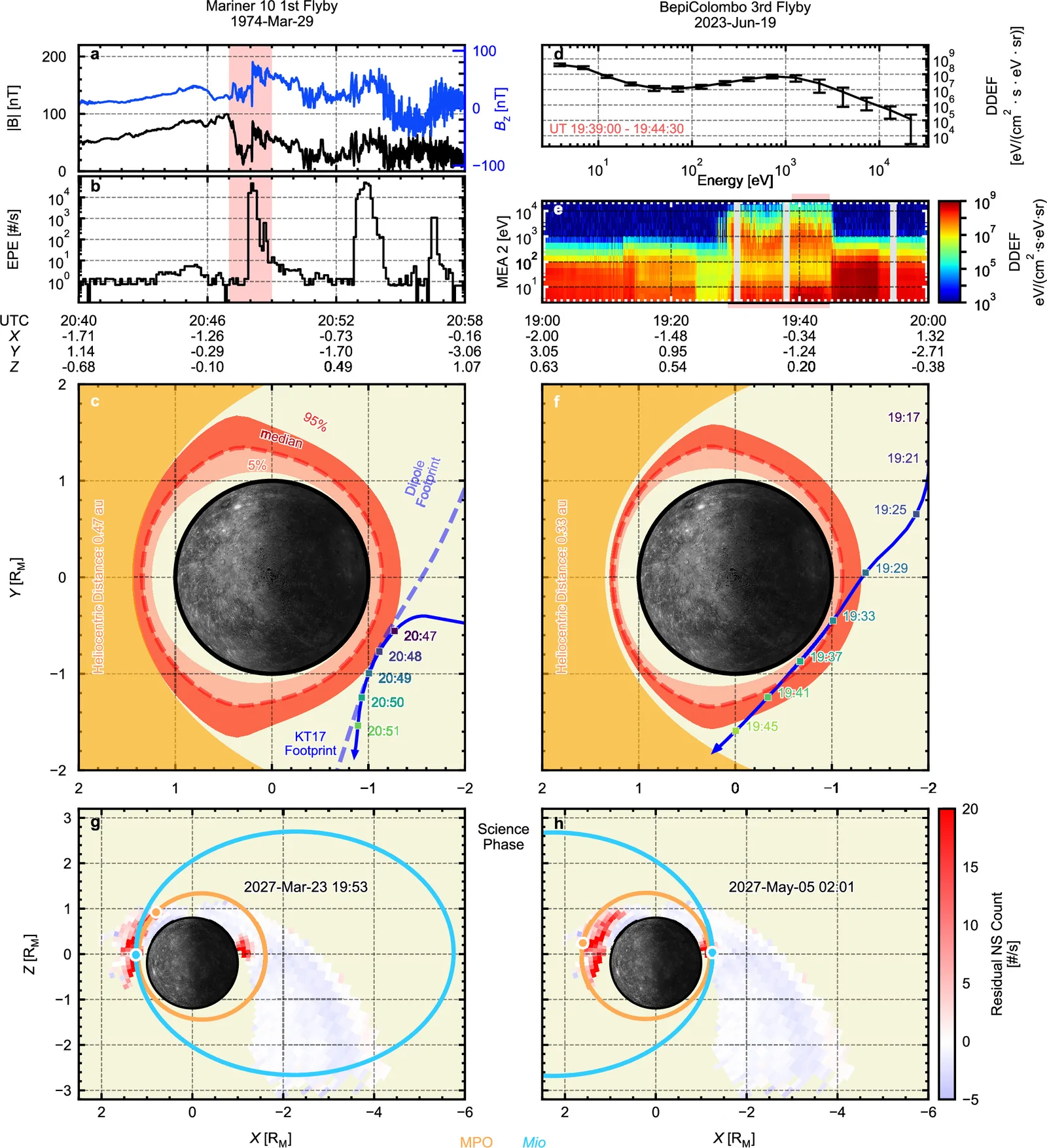
Energetic electron observations at Mercury from flybys. Left: Mariner 10. Right: BepiColombo. (**a**) Magnetic field strength (black) and Z component (blue). (**b**) Energetic Particles Experiment (EPE) > 35 keV electron count rate. (**c**, **f**) Equatorial footprints of the spacecraft during the events, overlaid on simulated electron trapping region boundaries. The red-shaded regions represent the statistical distribution of test-particle equatorial footprints; their inner edge, central line and outer edge correspond, respectively, to the 5th, 50th and 95th percentiles of the trapped electrons’ radial distance from Mercury. In the simulations, electrons were released from the midnight magnetosphere and traced within the KT17 model magnetic field with *r*_hel_ as input, corresponding to 0.47 au and 0.33 au for the flybys of Mariner 10 and BepiColombo. (**d**) The average electron energy spectrum (Directional Differential Energy Flux, DDEF) measured by Mercury Electron Analyzer (MEA). Here, error bars denote Poisson uncertainties. (**e**) MEA electron spectrum. (**g**) and (**h**) Two exemplary day-midnight plane orbits for BepiColombo MPO and Mio in their upcoming science phase in early 2027, overlaid with the distribution of average NS electron counts within the same format as in Fig. 3e. Mercury USGS/NASA MESSENGER MDIS basemap data in **c**,**f**–**h** from refs. ^64–66^.

Detailed radiation-belt dynamics at Mercury, including the continuous energization process for stably trapped electrons and the energy-sensitive non-adiabatic loss, await exploration in the upcoming BepiColombo era^40^. BepiColombo, slated to begin its two-spacecraft science mission at Mercury in 2027, may have already crossed Mercury’s radiation belt during its fly-bys of the planet (Extended Data Fig. 6d). Such dedicated measurements will shed further light on the intricate dynamics and variability of this extreme energetic particle system^41–43^.

## Methods

### MESSENGER Magnetometer

The flux-gate magnetometer measures the local vector magnetic field at a nominal 0.05-s cadence^44^.

### MESSENGER Fast-Imaging Plasma Spectrometer

The plasma mass spectrometer measures ions with energy per charge *E*/*q* = 0.046–13.6 keV *e*^−1^ and mass per charge 1–40 amu *e*^−1^ (ref. ^45^). It completes a sweep of its *E*/*q* steps at a nominal 10-s scan time within the magnetosphere.

### MESSENGER GRS and NS

The NS and GRS provided indirect measurements of energetic electrons (>10 keV)^46^. Impinging energetic electrons were stopped by the instrument housing and produced bremsstrahlung X-rays that reached borated-plastic (BP) scintillators and were read by optically coupled photomultiplier tubes, which incremented energy-resolved and marginal rate counters. Energetic electrons appear as temporary rate enhancements, particularly in the lower energy channels, superposed on a background dominated by planetary neutrons and galactic cosmic rays^16–18^. NS also collected coincidence and time-correlated data from which rates of incident fast planetary neutrons and galactic cosmic rays can be proxied^47^.

The NS and GRS BP energy channels were uncalibrated. However, both contain a spectral peak at an electron-equivalent energy of 93 keV from the ^10^B(n,*α*) → ^7^Li neutron capture reaction^16^. In the 64-channel NS spectra, this peak is located about channel 16. In the 1,024-channel GRS spectra, two such peaks exist, as the BP material is divided into two components but read by a single photomultiplier tube.

NS operated throughout the MESSENGER orbital mission at a nominal 20-s resolution near the planet (altitudes <6,000 km). GRS provided a 10-ms marginal rate and a 20-s energy-resolved rate beginning in March 2013 at altitudes <4,000 km. Before this, from the start of the orbital mission in March 2011 until the cryo-cooler failed in June 2012, GRS instead provided a lower resolution 60-s energy-resolved rate. The 10-ms rate was not collected close to the planet (<500-km altitude) onwards from September 2014.

We used these GRS and NS observations due to their greater sensitivity compared with measurements by the Energetic Particle Spectrometer. Both NS and GRS have geometric factors >10,000 times larger than those of the Energetic Particle Spectrometer^45,46^, which resulted in an orders-of-magnitude greater detection of energetic electron events^16^. Furthermore, the 10-ms GRS data are the fastest particle measurements collected by MESSENGER and best resolve the time dynamics of Mercury’s energetic electrons.

We used NS data at resolution ≤20 s and GRS data when the 10-ms rate was available. We performed minor data filtering (for example, removing anomalously low-count records) and removed orbits with <30 min of magnetosphere coverage^48^. To ensure that the energetic electrons analysed were accelerated by Mercury’s magnetosphere, we also excluded orbits containing solar energetic particle events or elevated populations of suprathermal solar wind electrons. We removed orbits that had a median high-altitude (>2*R*_M_) NS BP rate enhanced >30% compared with an expected value derived from fitting against heliocentric distance. Finally, we downsampled the small fraction of <20-s resolution NS data to 20-s to standardize the dataset and avoid artefacts in the subsequent data processing. This filtering resulted in 782,288 min (212,788 min) of NS (GRS) observations over 3,568 orbits (1,963 orbits).

### Detection algorithms for GRS and NS energetic electron events

We developed new event detection algorithms to provide the most robust, statistically confident and consistent identification of energetic electrons in GRS and NS BP data to date. These algorithms were based on previous approaches^16–18,49^ and capitalize on the bremsstrahlung and Poisson characteristics of the energetic electron signal in the BP scintillators. The algorithms differ only slightly between NS and GRS due to data resolution.

The main algorithm steps are:

1. Define the background rate by fitting the high-channel (electron-free) rate to the marginal rate.
2. Subtract this background from the marginal rate and convert the residual, detrended rate into a signal-to-noise ratio (SNR).
3. Identify energetic electron events as intervals with an enhanced, statistically significant SNR.

Previous approaches had specific limitations: the sliding-window method of ref. ^18^ misinterpreted long-duration, smooth-profile events (for example, Fig. 1f(i)) in the 10-ms GRS data as background, whereas ref. ^16^ was biased against short-duration events through its higher SNR requirements for single-record NS events. To mitigate these biases and to increase the sensitivity and robustness of our detection algorithms, we introduced several new features:

- Boxcar averaging over several timescales was applied to the SNR so that we could more completely sample the event amplitude versus duration parameter space. To maintain the highest timing precision, we identified events at their lowest smoothing possible.
- A high level of iteration throughout the algorithms (for example, updating the background after identifying event intervals) helps to ensure stable event identification and reduces the dependence on user-specified parameters.
- We defined a cost function to optimize the high-channel range used for defining the background. We thereby maximized the event signal while minimizing the background misfit and uncertainty, which improves event detection, particularly those of lower signal strength.
- The statistics-driven approach incorporates and propagates uncertainty at each step. This biases against false event detection (for example, a poor or uncertain definition of the background naturally raises the threshold to be considered an event).

We applied these algorithms to NS and GRS data for each orbit. The algorithms identified 4,610 NS and 5,596 GRS energetic electron events. Example algorithm outputs are shown in Supplementary Fig. 1 for the MESSENGER orbits shown in Fig. 1. The GRS algorithm found that the long-duration, variable-profile event recorded by NS in orbit 3791 comprised over two dozen distinct seconds-long events, and it confirmed that the event in orbit 2573 was indeed a single, continuous interval of energetic electrons.

### Classification of GRS and NS energetic electron events

We used signal analysis to identify stably drifting, spatially organized energetic electron events. We expected such structures to persist for magnetospherically extended durations (longer than a Dungey cycle), occur on closed field lines outside the bounce loss cone and have smooth SNR profiles. Although short-duration crossings and dynamics within a spatial structure (for example, wave–particle interactions) are possible, they are difficult to confidently distinguish from transient populations, particularly in the NS data. Our requirements were, therefore, conservative, and corresponding events reflect the clearest examples of stably trapped energetic electrons within the magnetosphere.

To identify such events, we first grouped events that are separated by <1 min, as events on such short timescales may be physically related^50^. This grouping of nearby events also helped to accentuate the short-scale signal variability, reconcile differences between GRS and NS event lists due to their different time resolutions, and correct for any potential artificial breaks produced by the detection algorithms in an otherwise continuous event (for example, due to stochastic variation). We then applied a fast Fourier transform (FFT) and band-pass analysis. For the band-pass analysis, we adapted the sliding-window approach from ref. ^18^ to decompose the SNR into a low-pass and high-pass signal. We summarized the properties of the FFT spectra and the SNR components (for example, median and mean), fitted the low-pass signal with a high-degree polynomial and examined the high-pass signal for statistically significant peaks.

Extended Data Fig. 2 shows examples of the band-pass analysis applied to events in Fig. 1. The low-pass signal accounts for nearly the entire SNR (97.6% for NS and 99.5% for GRS) in orbit 2573. There is no evidence in the high-pass component for short-lived signals (for example, injections). By contrast, the high-pass signal was dominant in orbit 3791. Although the low-pass signal was minor in GRS data (22.1%), the merging of individual events within the lower resolution NS data generated a more substantial low-pass contribution (40.6%).

Next, we classified GRS events, as these high-resolution observations best reveal short-lived signatures. We selected event groups with durations >3 min and considered the FFT, band-pass analyses and time series (for example, Fig. 1 and Extended Data Fig. 2) to label events as stable (for example, orbit 2573) or not (for example, orbit 3791). We then constructed a class definition by applying an ensemble clustering approach to these labelled events. This approach considers all two-parameter combinations of event, FFT and band-pass analysis features. Within each two-dimensional space (cluster), up to two linear divisions were used to optimally separate stable events by maximizing the Heidke skill score (HSS). Events were then scored based on this two-feature optimization. The lowest performing clusters were removed, and the event scores from across the top half of the remaining clusters were averaged. We set an average score threshold by maximizing the HSS. From the top 28 GRS clusters, a score threshold of 0.75 maximized the HSS at 0.975, corresponding to a probability of detection of 99.3%, a false discovery rate of 1.4% and an accuracy of 98.8%. Extended Data Fig. 3 illustrates example clusters and the distribution of average scores. These performance metrics and well-separated average scores demonstrate the success of this approach.

We extended this GRS class definition to NS events by repeating the same ensemble clustering. For each NS event group with duration >3 min that had high-resolution GRS observations available, we applied the GRS class definition to label the corresponding NS events as stable or not. We then repeated the ensemble clustering using the labelled NS events, the NS event features, the FFT features and the band-pass features. From the top 45 clusters, a score threshold of 0.80 resulted in HSS = 0.870, a probability of detection of 94.7%, a false discovery rate of 5.5% and an accuracy of 93.7%. We then applied this trained classification to all NS event groups. We overwrote the classification with direct GRS labelling when available to reduce the number of false positives and false negatives (<1% of events were overwritten). Finally, we applied spatial requirements to the NS events to complement the signal classification. As a fraction of SNR, we required >95% to be within the magnetosphere^48^, and of the portion within the magnetosphere, >95% to be on closed field lines outside the bounce unstable zone (see ‘Bounce unstable zone’ in Methods). These requirements removed <2% of stable-classified NS events.

### Aberrated MSM coordinate system

We present all observations with the aberrated Mercury solar magnetospheric (aMSM or ${{\rm{MSM}}}^{{\prime} }$) coordinate system. These coordinates are centred on Mercury’s magnetic dipole, which is well described where MESSENGER sampled the field as a dipole shifted northwards of the planetary centre by ~484 km or 0.2*R*_M_ where *R*_M_ = 2,440 km is Mercury’s radius^51^. In these coordinates, the *Z* axis points northwards parallel to the dipole axis. The *X* axis points antiparallel to the incoming solar wind, which is aberrated from the anti-Sunward direction from Mercury’s orbital motion about the Sun. We assumed a solar wind speed of 400 km s^−1^ to calculate the aberration angle (typically ~6°). Finally, the *Y* axis completes the right-handed system.

### Radiation-belt probability model

Direct MESSENGER crossings of stable energetic electron structures resulted in only a lower bound for their lifetimes. In many instances (Fig. 2), several orbits in a row had these structures. Without continuous observation, we cannot tell directly whether a structure lasted for this entire time or was an intermittent feature of the magnetosphere. To place some upper limits on structure lifetime, we applied the following statistical model:

1 $$f(N,P)=(1-P){P}^{N-1}$$

which describes the probability density function *f* of observing a radiation belt for *N* consecutive orbits in a row, assuming that the occurrence of the radiation belt is random with probability *P* and that each MESSENGER magnetosphere pass is independent (the radiation belt lasts for less than an orbit).

### NS energetic electron spectra

To remove the planetary neutron and galactic cosmic ray background from the NS BP spectra, which serve as a proxy for electron energy, for each time *t* in the NS time series, we:

1. Select the closest electron-free records before and after *t*.
2. Interpolate the count rate of each channel in these records to *t*.
3. Scale the total interpolated rate to the background rate provided by the NS algorithm.
4. Subtract this background spectrum.

Uncertainties (Poisson statistics and uncertainty in the background) were propagated.

### KT17 magnetic field model

We used the KT17 magnetic field model, a semi-empirical representation of Mercury’s internal and magnetospheric magnetic field, to provide a realistic magnetic environment for our simulation^52,53^. The KT17 model was developed based on magnetic field measurements from MESSENGER’s ~4,000 orbits and incorporates an offset axial dipole as well as contributions from magnetospheric currents. It demonstrates strong agreement with observed magnetic field data, with a root-mean-square residual of ~20–30 nT (under 20% of the total intensity; see Supplementary Fig. 3). As one of the most accurate models of Mercury’s magnetic field, KT17 is suited well for our study, despite certain limitations, which are discussed in the previous literature^23^.

An illustration of the KT17-modelled magnetic field line and intensity profile is presented in Extended Data Fig. 1. In this figure, we focus on the dayside magnetic field profile, which determines the morphology of the drift path of radiation-belt particles.

This model has two control parameters: the heliocentric distance of Mercury *r*_hel_, which predominantly modulates the solar wind pressure, and the disturbance index, which ranges from 0 (quiet) to 100 (active). In this study, we adjusted only the first parameter, and the disturbance index was fixed at 50, corresponding to a moderate state of the magnetosphere.

The solar wind dynamic pressure *p*_dyn_ was set in this model to be proportional to the inverse square of the heliocentric distance ${r}_{{\rm{hel}}}^{-2}$. This parameter scales the modelled magnetic field by modifying the position and intensity of the magnetopause current (equation (16) in ref. ^52^). Consequently, a higher *p*_dyn_ leads to a stronger induced magnetic field from the magnetopause, a more pronounced local maximum in the equatorial magnetic field and a more rapid transition from conventional to bifurcated bounce paths for drifting particles. These expected features are successfully reproduced by the KT17 model, as illustrated in Extended Data Fig. 4.

### Bounce unstable zone

The bounce unstable zone, illustrated in Extended Data Fig. 1, is in the northern hemisphere above the surface where conjugate magnetic fields in the southern hemisphere are below the planetary surface due to the northwards offset of the dipole centre (~0.196*R*_M_). This zone was identified by determining whether the position (*X*, *Y*, *Z*) in the northern hemisphere was above the planetary surface while its conjugate point (*X*, *Y*, −*Z*) in the southern hemisphere lies within the planetary volume:

2 $$\sqrt{{X}^{2}+{Y}^{2}+{\left[\pm Z-(-0.196{{\rm{R}}}_{{\rm{M}}})\right]}^{2}} < 1\,{{\rm{R}}}_{{\rm{M}}}$$

Extended Data Fig. 1 shows a schematic figure of the bounce unstable zone when local equatorial maxima are present (a bifurcated bounce path) and when they are absent (conventional bounce path).

Electrons within the bounce unstable zone that undergo inter-hemispheric bouncing will precipitate onto the southern surface, where the magnetic field intensity is weaker than the northern surface. Electrons within the bounce unstable zone that undergo intra-hemispheric bouncing can survive through the current drift cycle. This bounce unstable zone is used only for filtering the energetic electron events in MESSENGER observations. The simulations in our paper naturally include this effect due to the northwards offset of the dipole centre.

### Guiding centre simulation

The guiding centre simulations in this study were conducted with the KT17 magnetic field model.

#### Particle initialization

All test-particle parameter settings, including particle kinetic energy, pitch angle, radial distance and heliocentric distance, which modifies the KT17 model, are listed in Extended Data Table 1. Test particles were randomly released into the midnight section of the equatorial magnetosphere at the beginning of each simulation. Their energies and pitch angles were uniformly distributed within the parameter ranges. This ensured that the test-particle ensemble crossed the bifurcation separatrix with diverse bounce geometries, making the simulation results more representative of realistic radiation-belt populations.

#### Particle tracing

We used the guiding centre algorithm proposed by ref. ^54^ to trace the particles. In this algorithm, the guiding centre velocity of a particle is updated by the following equations:

3 $$\frac{{\rm{d}}{\bf{X}}}{{\rm{d}}t}=\frac{{p}_{\parallel }}{\gamma m}\frac{{{\bf{B}}}^{* }}{{B}_{\parallel }^{* }}+\frac{\mu }{\gamma q}\frac{{\bf{b}}\times \nabla B}{{B}_{\parallel }^{* }}$$

4 $$\frac{{\rm{d}}{p}_{\parallel }}{{\rm{d}}t}=-\frac{\mu }{\gamma }\frac{{{\bf{B}}}^{* }\cdot \nabla B}{{B}_{\parallel }^{* }}$$

where **X** is the position and *p*_∥_ is the momentum along the field line direction (**b** ≔ **B**/*B*) of the guiding centre. *γ*, *m* and *q* represent the Lorentz factor, rest mass and charge of the particle, respectively. *μ* ≔ *p*^2^/2*mB*_m_ denotes the magnetic moment of the particle. The modified magnetic field B^*^ in these equations can be derived from the modified electromagnetic potential A^*^, which are defined as follows:

5 $${{\bf{A}}}^{* }:= {\bf{A}}+\frac{{p}_{\parallel }}{q}{\bf{b}}$$

6 $${{\bf{B}}}^{* }:= \nabla \times {{\bf{A}}}^{* }={\bf{B}}+\frac{{p}_{\parallel }}{q}(\nabla \times {\bf{b}})$$

The modified magnetic field intensity, ${B}_{\parallel }^{* }:= {\bf{b}}\cdot {{\bf{B}}}^{* }=B+$ $({p}_{\parallel }/q)[{\bf{b}}\cdot (\nabla \times {\bf{b}})]$, incorporates corrections for the magnetic field shear and twist.

All the aforementioned magnetic field properties, including **B** and related gradient terms (for example, ∇ *B*, ∇ × **b** and **b** ⋅ ∇ × **b**) can be derived directly from the KT17 magnetic field model.

The simulation had a time step of 10^−4^ s, ~1/100 of the bounce period for an electron with an energy of 520 keV (which was the highest energy in our simulation). For comparison, the electron cyclotron period in a 200-nT magnetic field is ~2 × 10^−4^ s, implying that a time step of ~10^−6^ s (1/100 of the gyroperiod) would be needed to resolve full gyromotion in a fully kinetic simulation.

#### Particle loss evaluation

Particle loss was determined by monitoring both the electron position and the local magnetic field geometry. Electrons were considered lost if they crossed the magnetopause (magnetopause shadowing) or entered the planetary body (precipitation). Additionally, when the gyroradius of a particle (${r}_{g}:= \gamma m{v}_{\perp }/qB=\sqrt{2\mu mB}/qB$) exceeded 1/9 of the local magnetic field curvature radius *R*_c_, that is, $\kappa := \sqrt{{R}_{c}/{r}_{g}} < 3$, the guiding centre approximation breaks down, and so the particle was removed from tracking due to curvature-driven scattering^55^. The efficiency of curvature scattering was guaranteed because the electron gyroperiods remained of the order of 10^−4^ s to 10^−3^ s for magnetic field intensities between 10 nT and 100 nT. This is considerably shorter than their drift periods, which ranged from 10 s to 100 s (ref. ^17^). In such cases, particles were considered precipitated. This treatment probably overestimated the true loss rate but ensured numerical stability and is physically plausible within the guiding centre approximation.

The total number of particles lost in each simulation run was categorized by the underlying loss mechanism, as shown in Extended Data Fig. 5. The results indicate that magnetopause shadowing is comparable with curvature scattering, either under high *p*_dyn_ near perihelion or when particle initial drift shells are located further outwards radially. Both of these situations result in the drift orbit being closer to the magnetopause, confirming that the enhancement of magnetopause shadowing is physically consistent.

### Energetic electron measurements from other mercury missions: Mariner 10 and BepiColombo

Measurements from Mariner 10 and BepiColombo provide two rare but complementary snapshots of Mercury’s radiation environment (Extended Data Fig. 6). During Mariner 10’s first fly-by on 29 March 1974 (*r*_hel_ ≈ 0.47 au), its Energetic Particles Experiment (‘The Energetic Particle Experiment, Mercury Electron Analyzer’ in Supplementary Information) recorded several enhancements of >35-keV electrons^12^. Some of the high-energy (>300 keV) electron signals were later attributed to proton contamination and pulse-pileup effects^56^, but the first enhancement (shaded regions in Extended Data Fig. 6a,b) is considered reliable^13^. The first enhancement of energetic electrons lasted about 2 min and coincided with a magnetic dipolarization event. Mapping the spacecraft trajectory onto the modelled electron-trapping configuration (obtained from test-particle simulations at 300 keV, which is an upper limit for the energetic electrons observed by Mariner 10^13^) indicates that Mariner 10 intersected the outer edge of the trapping region (Extended Data Fig. 6c). This indicates that Mariner 10 probably observed an injection due to the inductive energization associated with a substorm^18,57,58^. This event was observed in the dawnside magnetotail, consistent with the region where substorm dipolarization events most frequently occur at Mercury^24,25^. Nevertheless, the duration of the enhancement was much longer than a typical injection event and was also sufficient for the 30-keV electrons to complete their initial drift in a post-dipolarization, dipolar field^18^. In general, this event could have signified the early formation of the radiation belt, although the long-term loss signature remains unavailable.

More recently, BepiColombo observed energetic electrons during its third fly-by on 19 June 2023 near Mercury’s perihelion (*r*_hel_ ≈ 0.33 au)^59^. As the spacecraft traversed from the outer to the inner drift shell, the Mercury Electron Analyzer (MEA)^41^, which had an upper energy limit of 26 keV, detected a clear enhancement in electron counts (Extended Data Fig. 6e). This enhancement exhibited a sharp spatial boundary and little temporal variability, which indicates that BepiColombo may have traversed a trapped electron population. However, the instrumental energy range was largely limited. Without measurements of electrons at higher energies (>100 keV), a more in-depth interpretation of this event is difficult, as low-energy electrons experience weak loss rates and can be trapped for notable periods even though higher energy electrons are not.

The electron radiation-belt structure obtained in our study for Mercury spatially overlaps the expected trajectories of BepiColombo’s dual spacecraft in its upcoming science phase in early 2027, meaning they are going to experience the radiation from this belt as well^40^ (Extended Data Fig. 6g,h). Their energy, angular-resolved particle measurements^41^, high-frequency wave observations^37^ and upstream monitor capability are expected to shed further light on the structure and variability of Mercury’s radiation-belt electrons, particularly by checking direct particle accelerations and losses.

## Supplementary information

Supplementary Information Supplementary Figs. 1–3, ‘Example GRNS detection algorithm outputs’, ‘Drift orbit bifurcation theory’, ‘Discrepancies between the KT17 model and MESSENGER observations’ and ‘The Energetic Particle Experiment (EPE), Mercury Electron Analyzer (MEA)’.

Peer Review File

## Data Availability

MESSENGER data are available at https://pds-ppi.igpp.ucla.edu/and https://pds-geosciences.wustl.edu/. Mariner 10’s magnetic field data are available at https://pds-ppi.igpp.ucla.edu/. The high-cadence data from the Energetic Particles Experiment of Mariner 10 were digitized from Fig. 2a of ref. ^13^ (openly available at ref. ^60^). The nominal-cadence data are available from the NASA Space Science Data Coordinated Archive (PSFP-00039). BepiColombo MEA data from the third fly-by are available in ref. ^61^. The Mercury MESSENGER MDIS base map used in this study is publicly available from the USGS Astrogeology Science Center of the US Geological Survey (USGS) at https://astrogeology.usgs.gov/search/map/mercury_messenger_mdis_basemap_loi_global_mosaic_166m.

## Extended data

**Extended Data Table 1:** Input parameters for the test-particle simulations

Panel	Energy (keV)	Pitch Angle Range (deg)	Radial Distance (R_M)	Heliocentric Distance (au)
Fig. 1a1 (Trajectory + shell)	3000 ± 600	43	1.2	0.467
Fig. 1a2 (Trajectory)	3000 ± 600	55	1.2	0.307
Fig. 1a2 (Shell)	300 ± 60	55 ± 5.0	1.2	0.307
Fig. 4a (Adiabatic)	300 ± 60	43	1.2	0.467
Fig. 4b (Diffusion)	300 ± 60	60	1.2	0.387
Fig. 4c (Advection)	300 ± 60	85	1.2	0.387
Fig. 4e	300 ± 60	67.5 ± 2.5	1.2	Variable
Fig. 4f	300 ± 60	Variable	1.2	0.387
Fig. 4g	300 ± 60	67.5 ± 2.5	Variable	0.387
Fig. 4h	Variable	67.5 ± 2.5	1.2	0.387
Extended Data Fig. 6c	300 ± 60	Loss cone	1.0-1.6	0.466
Extended Data Fig. 6f	300 ± 60	Loss cone	1.0-1.6	0.326

.

## Code availability

FORTRAN and Python implementations of the KT17 magnetic field model can be found in ref. ^53^ and https://github.com/mattkjames7/KT17. The complete IDL codes for processing the energetic electron data from the GRS and NS are openly available via Zenodo at https://doi.org/10.5281/zenodo.19663490 (ref. ^62^). Python test-particle codes are openly available via Zenodo at https://doi.org/10.5281/zenodo.19475783 (ref. ^63^). A complete project description, a user manual, a demo and example outputs are available from these Zenodo depositories^62,63^.
