# Supplementary material for: Dynamic electron radiation belt at Mercury controlled by solar wind driving
Source: Nat Astron. 2026 Jul 27;10(10):1489–99. doi: 10.1038/s41550-026-02925-3 (PMC13672091; doi:10.1038/s41550-026-02925-3)
Supplement: Supplementary file 1 [file 41550_2026_2925_MOESM1_ESM.pdf]

nature astronomy

Supplementary information https://doi.org/10.1038/s41550-026-02925-3

Dynamic electron radiation belt at Mercury controlled by solar wind driving

In the format provided by the authors and unedited

Table of Contents

Example GRNS Detection Algorithm Outputs 2

Drift Orbit Bifurcation Theory 4

- **Adiabatic Invariant** 4
- **From Guiding-Center to Drift-Bounce Hamiltonian** 5
- **Non-Equatorial Particle (Small Pitch Angle)** 6
- **Equatorial Particle (Large Pitch Angle)** 9

Discrepancies between the KT17 Model and MESSENGER Observations 12

The Energetic Particle Experiment (EPE), Mercury Electron Analyzer (MEA) 13

Reference 14

Example GRNS Detection Algorithm Outputs

Supplementary Figure 1 shows example visualizations of the NS and GRS energetic electron detection algorithm outputs. The two orbits (2573 and 3791) correspond to the orbits shown in Figure 1 in the Main Text. Differences in data availability (i.e., time coverage) and stochastic variability are immediately apparent between NS and GRS. Despite the greater noise in GRS measurements, the statistics-driven algorithm successfully identifies distinct energetic electron events in the 10-ms GRS data about 08:50 UTC in orbit 3791 while NS observed only one continuous, but variable, enhancement at 20-s resolution. By comparison, in orbit 2573, both instruments and algorithms detect the same, long-duration event despite differences in data resolution. The GRS data confirm that this event in orbit 2573 is one continuous interval of energetic electrons, as the BP rate does not meaningfully fall back to background levels. Notably, this event was undetected in the 10-ms GRS algorithm developed by ref. [1]. That algorithm used a 1-min sliding window to define the background rate, and as a result, the smooth profile of the event was misinterpreted as the background. To the opposite effect, the previous NS algorithm (ref. [2]) would have identified the orbit 3791 events but with much longer durations due to that approach's more generous SNR definition (e.g., only record-to-record variability was evaluated for uncertainty) and event logic. These orbits highlight the necessity of high-resolution data to resolve energetic electron dynamics at Mercury and the value of the improved GRNS detection algorithms.

The panels to the right of each subplot highlight additional valuable features of these algorithms. The upper panel in each subplot shows the distribution of SNR during non-event times within each orbit. These are well described by Gaussian distributions (cyan fits), which enable direct determination of statistical significance. The NS (GRS) algorithm considers SNR $>4\sigma$ ($>5\sigma$) above the mean SNR ($\overline{\text{SNR}}$) to be considered an event. These thresholds were chosen to limit the rate of false positives to <1% of total events and differ between NS and GRS due to data resolution. The lower panel in each subplot shows the cost function used to select the high-channel range in defining the background. Differences between orbits highlight the higher electron energies in orbit 2573 compared to orbit 3791, and the value of a flexible channel selection instead of a fixed range.

Supplementary Figure 1. Four example GRNS algorithm outputs (from top to bottom). (left, in each subplot) Marginal BP rate (black) and background rate (magenta) with energetic electron events shaded by the primary boxcar averaging used to detect them (horizontal color bar). (right) (top) The distribution of non-event SNR (grey) fitted with a Gaussian (cyan) used to determine statistical significance, and (bottom) the cost function (black) used to select the lower bound of the high-channel range (dashed magenta). The insert in GRS orbit 3791 highlights the detection of individual short-lived events.

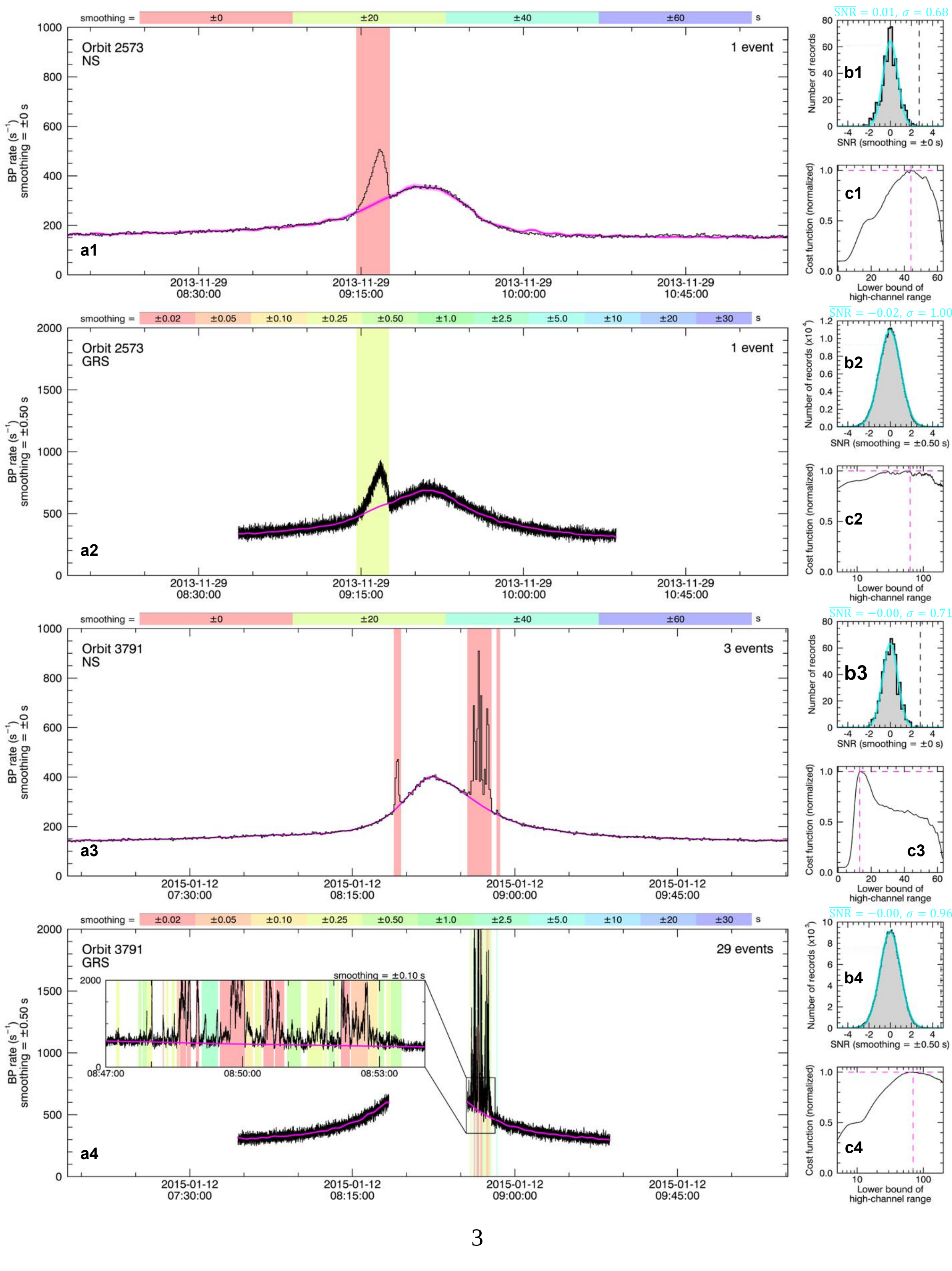
smoothing = ±0 ±20 ±40 ±60 s
Orbit 2573
NS
1 event
a1
BP rate (s⁻¹) smoothing = ±0 s
2013-11-29 08:30:00
2013-11-29 09:15:00
2013-11-29 10:00:00
2013-11-29 10:45:00
SNR = 0.01, σ = 0.68
b1
Number of records
SNR (smoothing = ±0 s)
c1
Cost function (normalized)
Lower bound of high-channel range
smoothing = ±0.02 ±0.05 ±0.10 ±0.25 ±0.50 ±1.0 ±2.5 ±5.0 ±10 ±20 ±30 s
Orbit 2573
GRS
1 event
a2
BP rate (s⁻¹) smoothing = ±0.50 s
SNR = −0.02, σ = 1.00
b2
Number of records (x10⁴)
SNR (smoothing = ±0.50 s)
c2
Orbit 3791
NS
3 events
a3
2015-01-12 07:30:00
2015-01-12 08:15:00
2015-01-12 09:00:00
2015-01-12 09:45:00
SNR = −0.00, σ = 0.71
b3
c3
Orbit 3791
GRS
29 events
smoothing = ±0.10 s
08:47:00
08:50:00
08:53:00
a4
SNR = −0.00, σ = 0.96
b4
Number of records (x10³)
c4

Drift Orbit Bifurcation Theory

In this section, we introduce how radial diffusion and advection can emerge from drift orbit bifurcation. This treatment is largely adapted from refs. [3, 4].

Adiabatic Invariant

Radiation belt electrons experience three fundamental periodic motions with well-separated periods: gyration, bounce, and drift. For a 100 keV electron in Mercury's magnetosphere, the periods of gyration, bounce, and drift are approximately 10^{-4}, 10^{-2}, and 30 seconds, respectively. This separation allows their evolution to be described using adiabatic invariant theory (refs. [5-7]), providing a zeroth-order description of radiation belt electron dynamics in the absence of rapid internal electromagnetic disturbances (e.g., whistler waves) or external variations.

The breakdown of the adiabatic invariant is closely associated with the particle's radial transport, which is the primary focus of this study. We emphasize that this association does not imply a causal relationship. Rather, both phenomena are manifestations of the underlying particle dynamics. However, adiabatic invariants are more amenable to theoretical generalization and analysis using established frameworks in mathematics and theoretical mechanics.

In this investigation, the bifurcation of the bounce path breaks the particle's bounce periodicity as well as the conservation of the **second adiabatic invariant** J, or alternatively, I when the particle's energy is conserved:

$$J \coloneqq \oint p_{\parallel} \mathrm{d}s = 2p \int_{s_1}^{s_2} \sqrt{1 - \frac{B(s)}{B_m}} \mathrm{d}s = 2pI \tag{1}$$

where $p(p_{\parallel}) \coloneqq \gamma m v(v_{\parallel})$ is the particle's relativistic momentum (component parallel to the magnetic field), and s is the particle's displacement along the magnetic field line. s_1 and s_2 are a pair of magnetic mirror points, where $B(s_1) = B(s_2)$ equals to the mirror point magnetic field intensity B_m of the particle. Magnetic mirror points s_1 and s_2 would alternate from inter-hemisphere to intra-hemisphere during the drift orbit bifurcation. As the magnetopause current intensifies, the equatorial magnetic field strength increases, and the particle's bounce periodicity gets disrupted. A particle's magnetic moment, $\mu \coloneqq p^2/2mB_m$, and B_m is also conserved as the gyro adiabaticity is not violated, and the kinetic energy is constant in a static magnetic field.

The conservation of energy is not only consistent with not including the electric field in our guiding-center tracing but also reasonable for a radiation belt particle with ~100s keV energy, whose energy is much higher than the energy changes due to the convection electric potential [8].

The radial transport (ΔR) is closely aligned with the change in I (ΔI) based on the relationship

$$\Delta R = \Delta I \cdot \left.\frac{\partial R}{\partial I}\right|_{B_m} \tag{2}$$

Where $\partial R/\partial I|_{B_m}$ is the partial derivative of the radial distance, R, versus I at a fixed B_m. This term is always positive in a dipolar region, as a particle away from the dipole center has a longer bounce path and therefore I.

From Guiding-Center to Drift-Bounce Hamiltonian

We start by evaluating this displacement, s, from the guiding center formalism (Equations A3 and A4 in the main text):

$$\frac{\mathrm{d}s}{\mathrm{d}t} \coloneqq \mathbf{b} \cdot \frac{\mathrm{d}\mathbf{X}}{\mathrm{d}t} = \frac{p_\parallel}{\gamma m} \tag{3a}$$

$$\frac{\mathrm{d}^2 s}{\mathrm{d}t^2} = \frac{1}{\gamma m}\frac{\mathrm{d}p_\parallel}{\mathrm{d}t} = -\frac{\mu}{\gamma^2 m}\frac{\mathbf{B}^*}{B_\parallel^*} \cdot \nabla B \approx -\frac{\mu}{\gamma^2 m}\frac{\partial B}{\partial s} \tag{3b}$$

where $\mathbf{X}$ is the position and $p_\parallel$ is momentum along the field line direction ($\mathbf{b} \coloneqq \mathbf{B}/B$) of the guiding center. γ, m, and q represent the Lorentz factor, rest mass, and charge of the particle, respectively. $\mu \coloneqq p^2/2mB_m$ denotes particle's magnetic moment. Modified magnetic field ($\mathbf{B}^*$) in these equations can be derived from the modified electromagnetic potential ($\mathbf{A}^*$) that is defined as follows:

$$\mathbf{A}^* \coloneqq \mathbf{A} + \frac{p_\parallel}{q}\mathbf{b} \tag{4}$$

$$\mathbf{B}^* = \nabla \times \mathbf{A}^* = \mathbf{B} + \frac{p_\parallel}{q}(\nabla \times \mathbf{b}) \tag{5}$$

The approximation $\mathbf{B}^* \approx \mathbf{B}$ adopted here is justified, as the change in the adiabatic invariant only occurs over a few bounce periods.

The magnetic field intensity along the field line can be expanded to its fourth order around the magnetic equator, where we set $s = 0$,

$$B(s; \mathbf{r_{eq}}) = B(0; \mathbf{r_{eq}}) + \frac{1}{2}s^2 \left.\frac{\partial^2 B}{\partial s^2}\right|_{\boldsymbol{s=0}} + \frac{1}{24}s^4 \left.\frac{\partial^4 B}{\partial s^4}\right|_{\boldsymbol{s=0}} + \mathcal{O}(s^6) \tag{6}$$

Here $\mathbf{r_{eq}}$ represents the magnetic equatorial footprint of the field line and will be omitted in the following text.

The odd terms in the expansion of B are omitted because they naturally become zero under the assumption that the magnetic field is symmetric around the magnetic equator, which is supported by the observation of Mercury's negligible tilt angle.

The equation of motion goes to

$$\frac{\mathrm{d}^2 s}{\mathrm{d}t^2} \approx -\frac{\mu}{\gamma^2 m}\frac{\partial B}{\partial s} = -\frac{\mu}{\gamma^2 m}\left[sB_{s=0}^{(2)} + \frac{1}{6}s^3 B_{s=0}^{(4)}\right] + \mathcal{O}(s^5) \tag{7}$$

Which can be formalized by the Hamiltonian

$$H(s, v_{\parallel}, t) = \frac{v_{\parallel}^2}{2} + \frac{\mu}{\gamma^2 m}\left[B(0) + \frac{1}{2}s^2 B_{s=0}^{(2)} + \frac{1}{24}s^4 B_{s=0}^{(4)} + \mathcal{O}(s^6) - B_m\right] \tag{8a}$$

$$= \frac{v_{\parallel}^2}{2} - \frac{\mu\left|B_{s=0}^{(2)}\right|}{2\gamma^2 m}s^2 + \frac{\mu B_{s=0}^{(4)}}{24\gamma^2 m}s^4 + \mathcal{O}(s^6) + \frac{\mu}{\gamma^2 m}[B(0) - B_m] \tag{8b}$$

$$\equiv 0 \tag{8c}$$

where $v_{\parallel} \coloneqq \dot{s} = p_{\parallel}/\gamma m$. $B_{s=0}^{(i)}$ denotes the i-th derivative of B versus s at the magnetic equator. Since we are concerned with the transition around the bifurcation point, where the magnetic field intensity has a local maximum along the field line, we get $B_{s=0}^{(2)} = -\left|B_{s=0}^{(2)}\right| < 0$.

This Hamiltonian is conserved, and its value is equal to the particle's kinetic energy per mass. The introduction of the constant term $-\mu B_m/\gamma^2 m$ ensures the Hamiltonian has a constant value of zero.

We define a non-conservative Hamiltonian, $\mathcal{H}$:

$$\mathcal{H}(s, v_{\parallel}, t) \coloneqq H(s, v_{\parallel}, t) - \frac{\mu}{\gamma^2 m}[B(0) - B_m] \tag{9a}$$

$$= \frac{v_{\parallel}^2}{2} + \frac{\mu}{\gamma^2 m}\left[\frac{1}{2}s^2 B_{s=0}^{(2)} + \frac{1}{24}s^4 B_{s=0}^{(4)}\right] + \mathcal{O}(s^6) \tag{9b}$$

The value of $\mathcal{H}$ is equal to $-\frac{\mu}{\gamma^2 m}[B(0) - B_m]$ as H is chosen to be constant zero (Equation 8c). Therefore, the sign of $\mathcal{H}$, reflects the topology of the bounce path: A positive (negative) $\mathcal{H}$ suggests the particle's B_m is stronger (weaker) than the equatorial intensity, $B(0)$, along the current magnetic field line, and the particle is bouncing between both hemispheres (within single hemisphere). This more practical non-conservative Hamiltonian, $\mathcal{H}$, will be the cornerstone of the following theoretical framework.

The parameters in both Hamiltonians, $B(0)$, $B_{s=0}^{(2)}$, and $B_{s=0}^{(4)}$, change slowly within individual bounce period as the particle drifts.

Non-Equatorial Particle (Small Pitch Angle)

The aforementioned Hamiltonians, $H(s, v_{\parallel}, t)$ and $\mathcal{H}(s, v_{\parallel}, t)$, can be simplified with variable substitution:

$$p = v_{\parallel}, \qquad q = \sqrt{\frac{\mu\left|B_{s=0}^{(2)}\right|}{\gamma^2 m}}s, \qquad \tau = \sqrt{\frac{\mu\left|B_{s=0}^{(2)}\right|}{\gamma^2 m}}t \tag{10}$$

Here, we get

$$H(q, p, \tau) = \frac{1}{2}(p^2 - q^2) + \frac{\mu}{\gamma^2 m}[B(0) - B_m] + \mathcal{O}(q^4) \tag{11}$$

And

$$\mathcal{H}(q,p,\tau) \coloneqq H(q,p,\tau) - \frac{\mu}{\gamma^2 m}[B(0) - B_m] = \frac{1}{2}(p^2 - q^2) + \mathcal{O}(q^4) \tag{12}$$

The q-based term is analogous to a conservative potential well $V(q) = -\frac{q^2}{2} + \mathcal{O}(q^4)$. Supplementary Figure 2 shows an example of the potential well and the Hamiltonian $\mathcal{H}$. Particles with sufficient energy, $p^2/2$, can pass through $q = 0$ and enter the opposite interval of q, forming an "inter-hemispheric bounce path". Each contour of $\mathcal{H}$ represents an instantaneous bounce trajectory. The transition from $\mathcal{H} < 0$ to $\mathcal{H} > 0$ reveals the occurrence of bifurcation. In the situation of the drift-bounce radiation belt particle, the change in $\mathcal{H}$ is caused by drift and is slow compared to the bounce frequency.

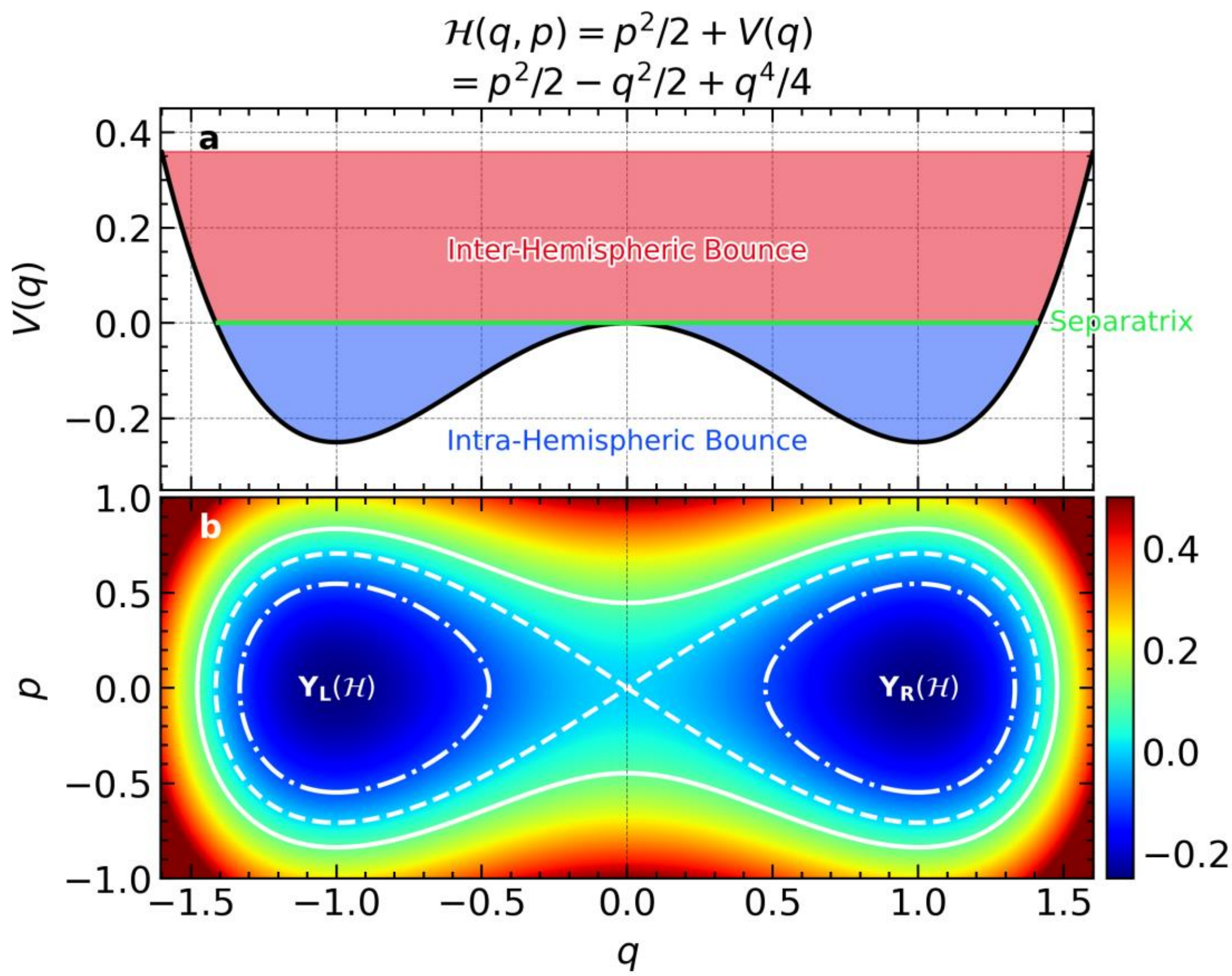

Supplementary Figure 2. An example plot of the potential well and Hamiltonian $\mathcal{H}$.(**a**) The black curve shows the potential well. (**b**) Contours of $\mathcal{H}$ according to this potential well. The dotted, dashed, and solid white contours correspond to $\mathcal{H} < 0$, $\mathcal{H} = 0$, and $\mathcal{H} > 0$.

The surrounding area enclosed by the contour lines of $\mathcal{H}$ is by definition the adiabatic invariant. Because this area systematically halves and doubles when the corresponding particle enters and exits the bifurcated bounce path, we note the surrounding areas in the positive and negative intervals of the q-axis Y_L and Y_R for simplifying the following discussion. In the symmetric magnetic field configuration that we considered, we have $Y_L = Y_R$ and both are noted as Y hereafter. For conventional (non-bifurcated) and bifurcated bounce paths, the adiabatic invariant $J = Y_L + Y_R = 2Y$ ($J = Y_L = Y_R = Y$).

$\mathcal{H}(q, p, \tau)$ satisfies the formulization requirements of separatrix crossing theory [9, 10]:

$$\mathcal{H}(q, p; \lambda) = \frac{\omega}{2}(p^2 - q^2) + \mathcal{O}(q^3, q^2 p, p^2 q, p^3, \lambda) \tag{13}$$

Where ω is a constant in the original formulation and signifies the characteristic frequency of the system near the saddle point, $p = q = 0$. It was chosen to be $\omega = 1$ in our coordinate system.

The separatrix crossing theory proves that the adiabatic invariant's changes due to the bifurcation are positively dependent on how fast the system of the bounce topology shifts, characterized by $\mathrm{d}Y/\mathrm{d}\tau$:

$$Y_1 = Y_0 - \frac{1}{\omega}\frac{\mathrm{d}Y}{\mathrm{d}\tau} \times \ln|2\sin\phi_0| = Y_0 - \frac{\mathrm{d}Y}{\mathrm{d}\tau} \times \ln|2\sin\phi_0| \tag{14}$$

Here $Y_0 = J_0$ and $Y_1 = 2J_1$ represent the single-lobe area before (subscript 0) and after (subscript 1) crossing the bifurcation separatrix, from conventional to bifurcated. The phase parameter ϕ_0 represents the bounce phase when the particle crosses the separatrix. It was demonstrated to be ergodic from 0 to 2π in numerical experiments. Thus, the ensemble average of $\ln|2\sin\phi|$, i.e., $\frac{1}{2\pi}\int_0^{2\pi} \ln|2\sin\phi|\,\mathrm{d}\phi = 0$, while the ensemble variance is finite ($\int_0^{2\pi} \ln^2|2\sin\phi|\,\mathrm{d}\phi \approx 5.17$). Thus, the change in action Y is **diffusive**.

The scaling factor from Y to I is constant and thus

$$\Rightarrow I_1 = \frac{I_0}{2} - \frac{1}{2}\frac{\mathrm{d}I}{\mathrm{d}\tau} \times \ln|2\sin\phi_0| \tag{15}$$

We define the

$$\Upsilon(\mathbf{r_{eq}}) \coloneqq 2\int_0^{s_m} \sqrt{1 - \frac{B(s)}{B(0)}}\,\mathrm{d}s \tag{16}$$

Here Υ is defined with the input of the field line equatorial footprints ($\mathbf{r_{eq}}$). This definition akin to that of I, in which the particle's intrinsic property, B_m, is replaced by a pure background magnetic field intensity at the magnetic equator. Near the separatrix, $B(0) \approx B_m$ and $\Upsilon(x, y) \approx I$. Furthermore, the time derivative of Υ is approximately equal to that of I and can be expressed as the product of the drift velocity $\mathbf{V_D}$ and the gradient of Υ.

$$\frac{\mathrm{d}I}{\mathrm{d}t} \approx \frac{\mathrm{d}\Upsilon}{\mathrm{d}t} \approx \langle \mathbf{V_D} \rangle_{\mathrm{B}} \cdot \nabla\Upsilon \tag{17}$$

Here $\langle \mathbf{V_D} \rangle_B$ denotes the bounce-averaged drive velocity as Υ is defined at the magnetic equatorial plane.

And finally, we get

$$I_1 = \frac{I_0}{2} - \frac{1}{2}\frac{\mathrm{d}I}{\mathrm{d}t}\frac{\mathrm{d}t}{\mathrm{d}\tau} \times \ln|2\sin\phi_0| \tag{18a}$$

$$= \frac{I_0}{2} - \frac{1}{2}\underbrace{\left[\sqrt{\frac{\gamma^2 m}{\mu\left|B_{s=0}^{(2)}\right|}}\left[\langle \mathbf{V_D}\rangle_b \cdot \nabla\Upsilon\right]\right]_{\ell_{b,0}} \times \ln|2\sin\phi_0|}_{\text{Diffusion}} \tag{18b}$$

In the same fashion, one can derive a similar result for the reversed process, i.e., the transition from the bifurcated bounce path to the conventional bounce path (noted by subscript 2):

$$I_2 = 2I_1 - \underbrace{\left[\sqrt{\frac{\gamma^2 m}{\mu\left|B_{s=0}^{(2)}\right|}}\left[\langle \mathbf{V_D}\rangle_b \cdot \nabla\Upsilon\right]\right]_{\ell_{b,1}} \times \ln|2\sin\phi_1|}_{\text{Diffusion}} \tag{19}$$

The above derivations explore the diffusive radial transport of the radiation belt particle after integrating the mapping relationship between I and radial distance R, i.e., Equation (2).

However, the above results based on the separatrix crossing theory are only theoretically validated when the particle's $I \gg r_g$, i.e., the small pitch angle radiation belt particle, as required by this theory. In the subsequent derivations, we are going to solve the situation for large pitch angle particles in an alternative way.

Equatorial Particle (Large Pitch Angle)

The evaluation still begins from the Hamiltonian, $\mathcal{H}(s, v_\parallel, t)$:

$$\mathcal{H}(s, v_\parallel, t) = \frac{v_\parallel^2}{2} + \frac{\mu}{\gamma^2 m}\left[\frac{1}{2}s^2\left|B_{s=0}^{(2)}\right| + \frac{1}{24}s^4 B_{s=0}^{(4)} + \mathcal{O}(s^6)\right] \tag{20}$$

Because the particle has a small bounce amplitude, s_m, the $\mathcal{O}(s^6)$ term is therefore omitted. Hamilton's canonical equations give that

$$\frac{\mathrm{d}^2 s}{\mathrm{d}t^2} = \frac{\mathrm{d}v_\parallel}{\mathrm{d}t} = -\frac{\partial H}{\partial s} = \frac{\mu\left|B_{s=0}^{(2)}\right|}{\gamma^2 m}s - \frac{\mu B_{s=0}^{(4)}}{6\gamma^2 m}s^3 \tag{21}$$

The coefficient, $\frac{\mu}{\gamma^2 m}\left|B_{s=0}^{(2)}\right|$, can be linearized as a function of time

$$\left|B_{s=0}^{(2)}\right| = \mathbf{V_D} \cdot \nabla\left|B^{(2)}\right| t \tag{22}$$

In which the origin of t is chosen to be the time $\left|B_{s=0}^{(2)}\right| = 0$.

By substituting variables

$$x = \sqrt[3]{a}t, \qquad y = \frac{\sqrt{\frac{b}{2}}}{\sqrt[3]{a}}s \tag{23}$$

Where

$$a = \frac{\mu}{m\gamma^2}\mathbf{V_D} \cdot \nabla\left|B^{(2)}\right|, \qquad b = \frac{\mu B^{(4)}}{6m\gamma^2} \tag{24}$$

The equation of bounce motion can be simplified in the form of the Painlevé-II equation:

$$\frac{\mathrm{d}^2 y}{\mathrm{d}x^2} = xy - 2y^3 \tag{25}$$

The dimensionless total area, S, surrounded by the curve (x, y) represents the adiabatic invariant with constant scaling from I.

$$S \equiv \oint \frac{\mathrm{d}x}{\mathrm{d}y}\mathrm{d}y = \frac{b}{2a}\oint \frac{\mathrm{d}s}{\mathrm{d}t}\mathrm{d}s = \frac{b}{a}vI \tag{26}$$

The general solution of the Painlevé-II equation is not analytically solvable, but its asymptotic properties at $x = \pm\infty$ can be used to diagnose the change in the adiabatic invariant. These asymptotic behaviors at $x = \pm\infty$ are suggestive because the phase space area at $x = \pm\infty$, $S_{\pm\infty}$ is equal to that just before (S_0) and after (S_1) the occurrence of bifurcation as the adiabaticity can be ensured.

And the change in the total area has been shown by previous literature (Its and Kapaev, 1988):

$$S_1 = S_0 - \frac{1}{2}\ln(e^{S_0} - 1) + \ln|2\sin\varphi| \tag{27}$$

$$S_2 = 2S_1 + \ln\left(2 + 2\sqrt{1 - e^{-S_1}}\sin\psi\right) \tag{28}$$

Let

$$\zeta \coloneqq \frac{a}{bv} = \frac{6}{vB^{(4)}_{s=0}}\mathbf{V_D} \cdot \nabla_\perp\left|B^{(2)}_{s=0}\right| \tag{29}$$

we have $S = I/\zeta$ and

$$I_1 = I_0 - \frac{\zeta}{2}\ln\left(e^{\frac{I_0}{\zeta}} - 1\right) + \zeta\ln|2\sin\varphi| = \frac{I_0}{2} \underbrace{- \frac{\zeta}{2}\ln\left(1 - e^{-\frac{I_0}{\zeta}}\right)}_{\text{Advection}} + \underbrace{\zeta\ln|2\sin\varphi|}_{\text{Diffusion}} \tag{30}$$

In addition to the diffusion term, $\zeta\ln|2\sin\varphi|$, the second term $-\frac{\zeta}{2}\ln\left(1 - e^{-\frac{I_0}{\zeta}}\right) > 0$, represents a positive term that is responsible for the radial advection.

The same knowledge can also be applied in the reverse process, yielding

$$I_2 = 2I_1 + \zeta\ln\left(2 + 2\sqrt{1 - e^{-\frac{I_1}{\zeta}}}\sin\psi\right) \tag{31}$$

For $I_0, I_1 \gg \zeta$, these two results degenerate to the diffusive behavior as expected by the evaluation in the previous section:

$$I_1 \approx \frac{I_0}{2} - \frac{\zeta}{2}\ln(1-0) + \zeta\ln|2\sin\varphi| = \frac{I_0}{2} - \underbrace{\zeta\ln|2\sin\varphi|}_{\text{Diffusion}} \tag{32}$$

$$I_2 = 2I_1 + \zeta\ln\left(2 + 2\sqrt{1-0}\sin\psi\right) = 2I_1 + \underbrace{\zeta\ln\left|2\cos\left(\frac{\pi}{4} - \frac{\psi}{2}\right)\right|}_{\text{Diffusion}} \tag{33}$$

By ignoring the higher-order terms in $B(s)$, the parameter Υ can be solved analytically:

$$\Upsilon = 2\int_0^{s_m}\sqrt{\frac{|B^{(2)}|s^2}{2B(0)} - \frac{|B^{(4)}|s^4}{24B(0)}}\,\mathrm{d}s = \frac{4\sqrt{2}\left|B^{(2)}\right|^{\frac{3}{2}}}{\sqrt{B_m}B^{(4)}} \tag{34}$$

$$\nabla\Upsilon = \frac{6\sqrt{2B_{s=0}^{(2)}}}{\sqrt{B_m}B_{s=0}^{(4)}} \cdot \nabla\left|B_{s=0}^{(2)}\right| \tag{35}$$

And the coefficient of the diffusion term can be given by

$$\frac{1}{2}\sqrt{\frac{\gamma^2 m}{\mu\left|B_{s=0}^{(2)}\right|}}\left[\langle\mathbf{V_D}\rangle_b \cdot \nabla\Upsilon\right] = \frac{1}{2}\sqrt{\frac{\gamma^2 m}{\mu\left|B_{s=0}^{(2)}\right|}}\left[\langle\mathbf{V_D}\rangle_b \cdot \nabla\Upsilon\right] \tag{36}$$

$$\frac{1}{2}\sqrt{\frac{\gamma^2 m}{\mu\left|B_{s=0}^{(2)}\right|}}\left[\langle\mathbf{V_D}\rangle_b \cdot \nabla\Upsilon\right] = \frac{1}{2}\sqrt{\frac{\gamma^2 m}{\mu\left|B_{s=0}^{(2)}\right|}}\,\frac{6\sqrt{2B_{s=0}^{(2)}}}{\sqrt{B_m}B_{s=0}^{(4)}} \cdot \nabla\left|B_{s=0}^{(2)}\right| = \frac{6}{vB_{s=0}^{(4)}}\mathbf{V_D} \cdot \nabla_\perp\left|B_{s=0}^{(2)}\right| = \zeta \tag{37}$$

Showing that both theories converge with each other at the small pitch angle end.

Discrepancies between the KT17 Model and MESSENGER Observations

We evaluated the absolute and relative residuals between the KT17 model and the observations using four metrics: root-mean-square (RMS, indicated by red squares in Supplementary Figure 3), upper quartile (top of the black error bars), median (middle circle), and lower quartile (bottom of the black error bars). These residuals were binned into five groups based on the magnetopause subsolar standoff distance for each corresponding orbit, which serves as a proxy for varying upstream dynamic pressure. When calculating the relative residuals, the observational data are restricted to radial distances of less than 1.5 Mercury radii (R_M) from the dipole center and measured magnetic field strengths (B_{obs}) greater than 100 nT. These criteria correspond to the inner dipolar region where radiation belt particles drift.

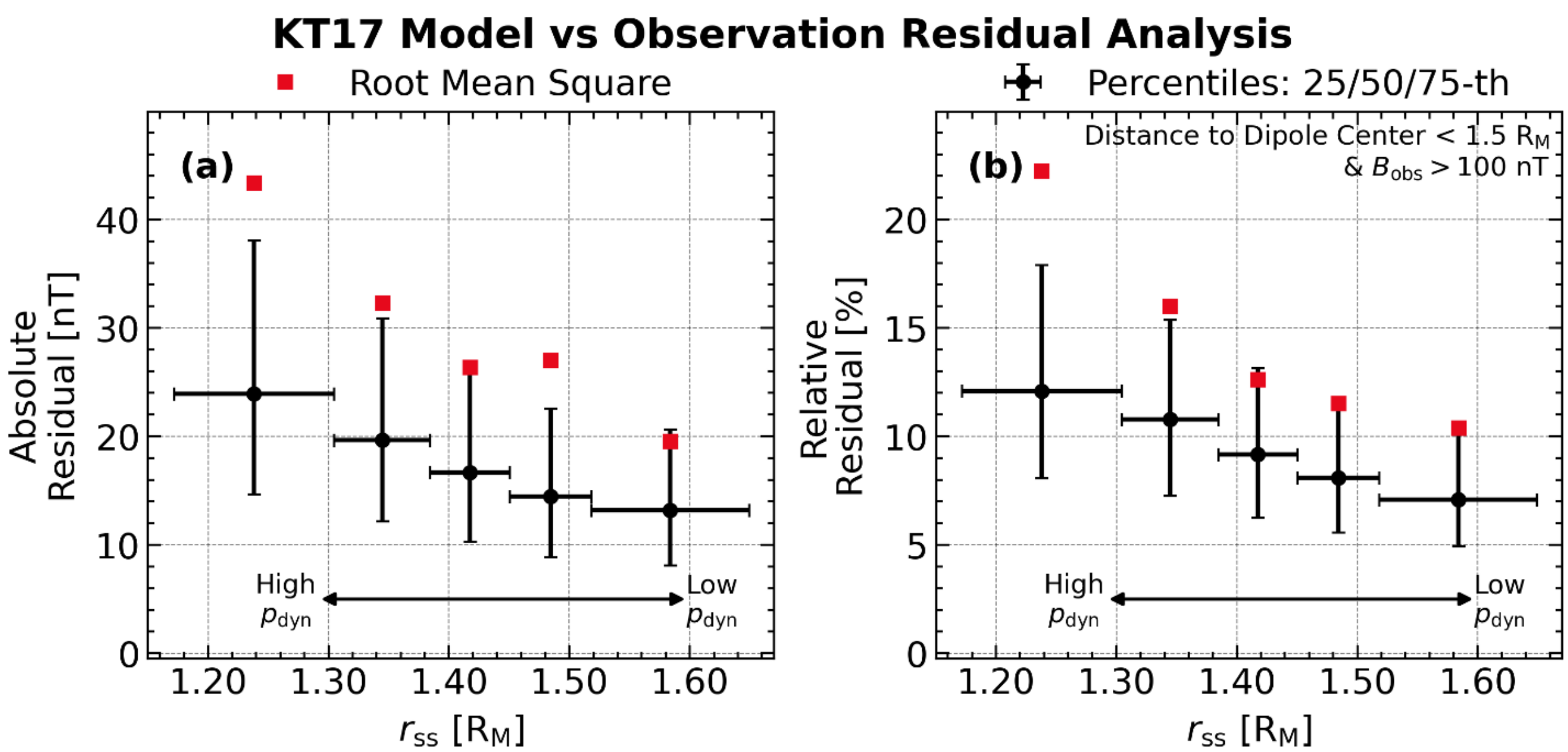

Supplementary Figure 3. Residual analysis of the KT17 model compared to observations. (**a**) Absolute residuals ($\delta B = |\mathrm{B}_{\mathrm{KT17}} - \mathrm{B}_{\mathrm{obs}}|$). (**b**) Relative residuals ($\delta B / B_{\mathrm{obs}}$). The red squares and vertical black error bars represent the root-mean-square and percentiles (25th, 50th, and 75th) of these residuals, respectively. Horizontal errors denote the width of each r_{ss} bin. Each bin contains data from over 700 orbits.

The analysis shows that both absolute and relative residuals tend to increase under stronger solar wind forcing. The RMS absolute residual reaches ~43 nT when $r_{ss} < 1.3\ \mathrm{R_M}$. However, the RMS relative residual within the dipolar region remains below 25%; hence, the model is still considered acceptable in the radiation belt region. Notably, the upper quartiles of the residuals are less than or comparable to the RMS values. This indicates that the RMS is likely inflated by a small percentage of large deviations, which could be attributed to polar cusp diamagnetic effects or field-aligned currents [11, 12]. Consequently, in about 75% of cases, the actual model-observation discrepancy is smaller than what the RMS residual implies.

The Energetic Particle Experiment (EPE), Mercury Electron Analyzer (MEA)

The EPE onboard Mariner 10, including the Main Telescope (MT) and Low-Energy Telescope (LET), were designed to measure energetic electrons and ions in Mercury's magnetosphere. The MT utilized pulse height analysis to detect electrons in the 0.2–50 MeV range, while the LET focused on lower-energy protons and helium nuclei. Both instruments provided high-time-resolution measurements at 0.6-second intervals, capturing rapid variations in charged particle flux.

The Mercury Electron Analyzer (MEA), part of the Mercury Plasma Particle Experiment (MPPE) onboard the Mio spacecraft of the BepiColombo mission [13, 14], is designed to study Mercury's plasma environment. MEA consists of two identical sensors (MEA1 and MEA2), each incorporating a top-hat electrostatic analyzer to measure low-energy electron distributions ranging from 3 eV to 26 keV with a nominal cadence of 0.25 Hz. These sensors feature a 360° field of view with an angular resolution of 22.5° × 11.25° and are capable of dynamically adjusting their geometrical factor by a factor of 1000, enabling precise detection of both solar wind and magnetospheric electron fluxes. With at most 64 contiguous energy channels, MEA performs full energy sweeps to capture detailed 3D electron distributions, playing a key role in analyzing Mercury's magnetosphere dynamics, solar wind interactions, and electron transport and acceleration processes, providing vital insights into planetary plasma physics. It is worth noting that the field of view of MEA was partially obstructed by the spacecraft's thermal shield during the cruise phase, which may have led to an underestimation of the observed electron flux.

Reference

[1] Dewey, R. M., Slavin, J. A., Raines, J. M., Baker, D. N. & Lawrence, D. J. Energetic electron acceleration and injection during dipolarization events in Mercury's magnetotail. *J. Geophys. Res. Space Phys.* **122**, 12170–12188 (2017).

[2] Lawrence, D. J. et al. Comprehensive survey of energetic electron events in Mercury's magnetosphere with data from the MESSENGER Gamma-Ray and Neutron Spectrometer. *J. Geophys. Res. Space Phys.* **120**, 2851–2876 (2015).

[3] Öztürk, M. K. & Wolf, R. A. Bifurcation of drift shells near the dayside magnetopause. *J. Geophys. Res. Space Phys.* **112**, A07207 (2007).

[4] Ukhorskiy, A. Y., Sitnov, M. I., Millan, R. M. & Kress, B. T. The role of drift orbit bifurcations in energization and loss of electrons in the outer radiation belt. *J. Geophys. Res. Space Phys.* **116**, A09208 (2011).

[5] Lyons, L. R., Thorne, R. M. & Kennel, C. F. Pitch-angle diffusion of radiation belt electrons within the plasmasphere. *J. Geophys. Res.* **77**, 3455–3474 (1972).

[6] Ozeke, L. G., Mann, I. R., Murphy, K. R., Rae, I. J. & Milling, D. K. Analytic expressions for ULF wave radiation belt radial diffusion coefficients. *J. Geophys. Res. Space Phys.* **119**, 1587–1605 (2014).

[7] Summers, D., Ni, B. & Meredith, N. P. Timescales for radiation belt electron acceleration and loss due to resonant wave-particle interactions: 2. Evaluation for VLF chorus, ELF hiss, and electromagnetic ion cyclotron waves. *J. Geophys. Res. Space Phys.* **112**, A04207 (2007).

[8] Jasinski, J. M., Slavin, J. A., Raines, J. M. & DiBraccio, G. A. Mercury's solar wind interaction as characterized by magnetospheric plasma mantle observations with MESSENGER. *J. Geophys. Res. Space Phys.* **122**, 12,153–12,169 (2017).

[9] Cary, J. R., Escande, D. F. & Tennyson, J. L. Adiabatic-invariant change due to separatrix crossing. *Phys. Rev. A* **34**, 4256–4275 (1986).

[10] Cary, J. R. & Skodje, R. T. Phase change between separatrix crossings. *Phys. D Nonlinear Phenom.* **36**, 287–316 (1989).

[11] Anderson, B. J. et al. Steady-state field-aligned currents at Mercury. *Geophys. Res. Lett.* **41**, 7444–7452 (2014).

[12] Dewey, R. M., Raines, J. M., Jasinski, J. M. & Slavin, J. A. Interpreting energy-latitude dispersions in Mercury's northern magnetospheric cusp with MESSENGER. *J. Geophys. Res. Space Phys.* **130**, e2025JA034278 (2025).

[13] Saito, Y. et al. Pre-flight calibration and near-Earth commissioning results of the Mercury Plasma Particle Experiment (MPPE) onboard MMO (Mio). *Space Sci. Rev.* **217**, 70 (2021).

[14] Sauvaud, J.-A. et al. The Mercury Electron Analyzers for the BepiColombo mission. *Adv. Space Res.* **46**, 1139–1148 (2010).
