# Supplementary material for: Dynamic electron radiation belt at Mercury controlled by solar wind driving
Source: Nat Astron. 2026 Jul 27;10(10):1489–99. doi: 10.1038/s41550-026-02925-3 (PMC13672091; doi:10.1038/s41550-026-02925-3)
Supplement: Supplementary file 2 [file 41550_2026_2925_MOESM2_ESM.pdf]

nature portfolio

Peer Review File

Dynamic Electron Radiation Belt at Mercury Controlled by Solar Wind Driving

Corresponding Author: Dr Weijie Sun

This manuscript has been previously reviewed at another journal. This document only contains information relating to versions considered at Nature Astronomy.

This file contains all reviewer reports in order by version, followed by all author rebuttals in order by version.

Version 0:

Reviewer comments:

Reviewer #1

(Comments for the Author)
This manuscript presents a high-level, detailed, and rigorous study of the radiation belt at Mercury. The authors effectively combine multiple datasets, statistical analyses, and state-of-the-art simulations to address the longstanding question of the presence or absence of a radiation belt within Mercury's compact and highly dynamic magnetosphere. Overall, the paper is well-structured and, in my view, suitable for publication in Nature Astronomy after minor revisions.

I have the following comments:

There appears to be a potential logical inconsistency in the use of the guiding center approximation—where the magnetic moment is assumed to be conserved—alongside the discussion of the kappa parameter, which is typically used to diagnose scattering and violations of magnetic moment conservation. It is possible that I am missing a key point in how the authors reconcile this issue, but additional clarification would be helpful for the reader.

The authors identify drift orbit bifurcation as a primary driver of particle losses; however, the discussion is limited to the classical symmetric framework (e.g., Shabansky, Ukhorskiy). While this model is valid, it assumes north–south and dawn–dusk symmetry in the magnetic field configuration, leading to a symmetric separatrix structure. In reality, Mercury's magnetic field is significantly asymmetric due to dipole tilt and interplanetary magnetic field (IMF) effects. Such asymmetries are expected to further disrupt adiabatic invariants and enhance particle losses. Recent studies (e.g., 10.1029/2024JA032987; 10.1029/2025GL116514) demonstrate that asymmetric configurations can strongly accelerate this process. I suspect this effect is particularly relevant for Mercury's radiation belt and encourage the authors to discuss it.

For planets with strong intrinsic magnetic fields (e.g., Earth, Jupiter, Saturn), radiation belt dynamics are largely governed by wave–particle resonant interactions, especially with whistler-mode waves. The authors' conclusions appear to suggest that such mechanisms are not significant at Mercury. This is an important point and should be explicitly stated and clarified in the manuscript.

Overall, this is a strong and timely contribution, and addressing the points above would further strengthen the paper.

(Remarks on code availability)

Reviewer #2

(Comments for the Author)
Zhao and Dewey et al. Dynamic electron radiation belt at Mercury controlled by solar wind driving. (2026)—> ZD26 hereafter.

ZD26 present observational evidence that Mercury's magnetosphere can magnetically confine and accelerate electrons

over long dynamical timescales. The confirmation of long-lived trapped energetic electrons—i.e., radiation belts—at Mercury would constitute a landmark result, demonstrating that stable particle trapping can persist even in one of the most extreme and strongly driven magnetospheric environments in the Solar System. Such a finding would challenge the prevailing assumption that strong solar wind forcing, small system size, and weak magnetic fields inherently preclude sustained trapping, and would therefore require a revision of current theoretical frameworks in plasma physics and magnetospheric dynamics. More broadly, this result would suggest that key processes—such as adiabatic invariance, wave–particle interactions, and radial transport—remain effective, or reorganize in ways not yet fully understood, in regimes far from those typically considered. The implications extend beyond Mercury, with direct relevance for how radiation belts are modeled at Earth, other planets, and substellar magnetospheres.

I do not have specific reasons to question the validity of the observational evidence derived from the gamma-ray spectrometer (noting that this is not a dataset I have personally worked with). The proposed theoretical interpretation of the transition between trapped and untrapped orbits—based on enhanced solar wind perturbations near perihelion—is physically plausible and consistent with expectations. However, in its current form, the theoretical framework is not presented with sufficient clarity or justification to fully support the authors' conclusions. I therefore recommend major revision and provide below specific suggestions to improve the manuscript, particularly regarding the physical interpretation of the loss of confinement under strong driving conditions.

Comments:

1. The authors propose that the loss of confinement is primarily driven by drift-orbit bifurcation (last sentence p.9). However, the relevant sections (in particular the Appendix) are difficult to follow and appear to conflate distinct physical processes. Specifically, mechanisms associated with particle loss (e.g., bounce-unstable orbits arising from drift-orbit bifurcation) are not clearly distinguished from those responsible for acceleration and transport (e.g., radial advection and diffusion, which may also be influenced by drift-orbit bifurcation). While all of these processes are in principle plausible, their respective roles are not sufficiently articulated or demonstrated for strong and weak driving conditions.

In addition, the implementation of the theoretical framework lacks clarity. The authors appear to use the guiding-centre formalism of Brizard et al. (PoP) to compute particle trajectories, but the underlying magnetic field model is not specified. It is unclear whether the field is derived from the KT17 empirical model, and if so, how this model is incorporated into the guiding-centre Hamiltonian. In particular, the form of the vector potential, and its parametrization as a function of solar wind dynamic pressure, are not described.

This raises a more fundamental issue regarding the consistency between the different theoretical ingredients used in the manuscript. It is not evident how the KT17 magnetic field model connects to the drift-orbit Hamiltonian framework of Öztürk and Wolf (JGR, 2007), which is invoked to interpret radial transport (diffusive and/or convective). While KT17 may be suitable to estimate the location of bounce-unstable regions (e.g., via Eq. A2), the dependence of the bifurcation parameters (e.g., the λ parameter) on the magnetic field configuration or on dynamical pressure is neither explained nor quantified.

More generally, although the Brizard formalism and the Öztürk and Wolf framework both originate from a Hamiltonian description, they are expressed in different variables. The manuscript does not make clear how these approaches are reconciled in practice. This lack of consistency, combined with the limited description of the numerical implementation, prevents a clear assessment of whether drift-orbit bifurcation alone can account for both the observed losses and the inferred acceleration of trapped electrons. Clarifying these points would significantly strengthen the physical interpretation and improve confidence in the proposed mechanism.

2. The authors understate the significance of their results in the Introduction. The potential discovery of radiation belts at Mercury would not only provide a distinct laboratory for studying space weather processes relevant to Earth, but also probe regimes that are largely inaccessible in the terrestrial magnetosphere. Mercury's magnetosphere operates under far more extreme conditions and may, in some respects, be viewed as an analogue of Earth's response to rare, highly disturbed Carrington-like events.

More importantly, the implications extend beyond the Earth–Mercury comparison. The existence of radiation belts at Mercury would directly inform outstanding questions on particle acceleration and transport across a wide range of planetary and substellar magnetospheres (DOI: 10.1126/sciadv.aea4945), which operate in regimes significantly different from Earth (e.g., strong vs weak co-rotation, synchrotron losses, stellar-wind- versus rotation-driven systems). As noted by the authors (footnote 2), the drift period of energetic particles at Mercury is comparable to the injection timescale, implying a system that is persistently driven far from local thermodynamical equilibrium. This is a qualitatively different regime from that typically assumed for Earth's radiation belts. In this context, Mercury could serve as a critical testbed for the validity of current quasilinear wave–particle acceleration models, including both local interactions and radial transport (DOI:10.3847/1538-4365/acff6a). These models generally rely on assumptions of weak departures from equilibrium and diffusive saturation of instabilities associated with loss and acceleration processes—assumptions that are unlikely to hold under Mercury-like conditions. This broader significance should be more clearly articulated, particularly for an astronomy and astrophysics readership that is often more focused on fundamental plasma processes in such environments than on space weather specifically.

3. As far as drift-orbit bifurcation is concerned, the authors may wish to consider the following recent study, which accounts for more realistic asymmetric potentials in the Hamiltonian and provides an estimate of the radial transport advection timescale that can be compared to their observations: DOI: https://doi.org/10.1103/nsg2-scpg.

4. Regarding Section A.9, the authors state that the KT17 model shows strong agreement with observed magnetic field data, citing a root mean square residual of 20–30 nT. However, this metric would be more informative if placed in the context of varying solar wind driving conditions. Given the large variability of Mercury's magnetic field strength across different regions and levels of external forcing, an absolute residual expressed in nT is difficult to interpret in terms of model accuracy. In particular, it is unclear whether a 20–30 nT discrepancy represents a small or significant deviation under weak versus strong driving conditions.

Providing a normalized measure of the residual (e.g., relative to the local magnetic field strength), or stratifying the error as a function of dynamical pressure, would help clarify the level of agreement and its relevance for the physical processes discussed. It should also be noted that, in the case of drift-orbit bifurcation, it is not only the magnetic field magnitude that matters, but also its gradients, and it is not clear to what extent this can or has been quantified for KT17.

(Remarks on code availability)
At present, no README or equivalent documentation appears to be provided for the Python codes, which makes it difficult to assess their intended usage, dependencies, and relation to the results shown in the manuscript. More importantly, the code used to process the gamma-ray spectrometer data—the crucial dataset underlying the identification of radiation belts at Mercury—is written in IDL. I was not able to evaluate this part of the workflow directly, in part because no documentation is provided and in part because IDL requires proprietary licensed software. As a result, the most critical step of the analysis is not readily reproducible from the materials currently described.

Version 1:

Reviewer comments:

Reviewer #1

(Comments for the Author)
Authors have addressed all my comments and i'm ready to recommend this paper for publication

(Remarks on code availability)

Reviewer #2

(Comments for the Author)
The authors have carefully addressed my previous concerns and suggestions, and the manuscript is now in excellent shape. However, the exchange with the other reviewer appears to have introduced a minor issue that I would ask the authors to revise.

The statement that radiation belt dynamics at Jupiter and Saturn are "largely governed by wave–particle resonant interactions, especially with whistler-mode waves" appears to be an overgeneralization from the terrestrial case and is not supported by the review cited by the authors (Roussos & Kollmann). At Earth, wave–particle interactions (e.g., chorus and hiss) indeed play a dominant role for electrons in the ~10 to hundreds of keV range and are central to both acceleration and loss processes. However, this framework does not directly extend to the giant planets.

The cited review emphasizes that Jupiter and Saturn operate in a fundamentally different regime, where magnetospheric dynamics are largely internally driven (due to rapid planetary rotation and strong plasma sources from moons), rather than solar-wind driven. As a result, several processes play a primary role in shaping the radiation belts, including:
(1) radial transport (both diffusive and convective) and associated adiabatic acceleration,
(2) interchange instability, which drives rapid inward transport of <MeV particles,
(3) corotation and large-scale electric fields, and
(4) strong coupling to moons, rings, and neutral material, which act as both sources and sinks.

Wave–particle interactions are discussed in the cited work, but not as the dominant controlling mechanism. For Saturn, local acceleration by whistler-mode chorus is described as inefficient, with electron populations primarily controlled by radial transport and loss processes. For Jupiter, chorus waves may contribute to energizing freshly injected particles but become inefficient once distributions reach steady state, while radial transport remains a key energy source.

Furthermore, for ions and for large portions of the electron populations at both planets, dominant processes include CRAND (especially for Saturn's MeV protons), radial diffusion, interchange injections, charge exchange, and absorption by moons and rings. These processes differ significantly from the standard Earth radiation belt paradigm and often dominate over wave-driven diffusion.

Therefore, the statement in its current form is misleading and should be revised. A more accurate formulation would be that wave–particle interactions are one of several contributing processes, while radial transport, corotation-driven dynamics, and moon–magnetosphere coupling play a central—often dominant—role at Jupiter and Saturn, depending on particle species and energy.

(Remarks on code availability)
README files are now provided and steps by steps details for installation are clearly explained.

Open Access This Peer Review File is licensed under a Creative Commons Attribution 4.0 International License, which permits use, sharing, adaptation, distribution and reproduction in any medium or format, as long as you give appropriate credit to the original author(s) and the source, provide a link to the Creative Commons license, and indicate if changes were made.
In cases where reviewers are anonymous, credit should be given to 'Anonymous Referee' and the source.
The images or other third party material in this Peer Review File are included in the article's Creative Commons license, unless indicated otherwise in a credit line to the material. If material is not included in the article's Creative Commons license and your intended use is not permitted by statutory regulation or exceeds the permitted use, you will need to obtain permission directly from the copyright holder.
To view a copy of this license, visit https://creativecommons.org/licenses/by/4.0/

Reply to the two reviewers

We thank both reviewers for their careful evaluation and constructive suggestions, which helped us in improving the study and the manuscript. Below includes a point-by-point response to all comments from the reviewers. A track-changed revised manuscript has also been included in the submitted documents.

Reviewers' comments:

Reviewer #1 (Comments for the Author):

This manuscript presents a high-level, detailed, and rigorous study of the radiation belt at Mercury. The authors effectively combine multiple datasets, statistical analyses, and state-of-the-art simulations to address the longstanding question of the presence or absence of a radiation belt within Mercury's compact and highly dynamic magnetosphere. Overall, the paper is well-structured and, in my view, suitable for publication in Nature Astronomy after minor revisions.

I have the following comments:

There appears to be a potential logical inconsistency in the use of the guiding center approximation—where the magnetic moment is assumed to be conserved—alongside the discussion of the kappa parameter, which is typically used to diagnose scattering and violations of magnetic moment conservation. It is possible that I am missing a key point in how the authors reconcile this issue, but additional clarification would be helpful for the reader.

We appreciate the opportunity to clarify the framework's consistency. The logic remains sound. However, we recognize that a more detailed description is required to preclude potential confusion:

We use the kappa parameter to identify instances within the particle-tracing simulations that violate magnetic moment conservation. Here, the gyro radius is calculated based on the guiding center's magnetic moment, μ, and the model magnetic field value at its position, $B(\mathbf{X})$, i.e.,

$$r_g := \frac{\gamma m v_\perp}{qB} = \frac{\sqrt{2\mu m B(\mathbf{X})}}{qB(\mathbf{X})}$$

Particles with kappa < 3 are expected to undergo rapid pitch angle scattering. This non-adiabatic motion violates the guiding center approximation and requires the particle to be

ejected from the simulation. We find that kappa < 3 only occurs in the magnetotail on closed field lines near the equatorial region (see **Figure 4c**). Although we stop tracing the particles, we consider them to be lost to the planetary surface since the curvature scattering on closed field lines would result in trajectories that precipitate to either planetary hemisphere. The kappa parameter, therefore, not only identifies where the guiding center approximation is violated, but also where test particles are going to be subjected to curvature scattering and be lost via precipitation.
To justify the resulting scattering, we considered the timescales involved. In a typical nightside magnetic field strength of 10 - 100 nT, the electron gyro-frequency ($eB/2\pi m_e$) ranges from 280 - 2800 Hz. As a comparison, the drift periods of 30-300 keV electrons are 10-100 seconds (Baker et al., 2016, also checked with our simulation results). Such a distinct difference in time scale ensures the electrons would be scattered sufficiently in at least hundreds of gyro-periods when the kappa values reach the non-adiabatic threshold of below 3.

We have summarized this discussion and added it to <u>**section A.11, Lines 954-961**</u>.

The authors identify drift orbit bifurcation as a primary driver of particle losses; however, the discussion is limited to the classical symmetric framework (e.g., Shabansky, Ukhorskiy). While this model is valid, it assumes north–south and dawn–dusk symmetry in the magnetic field configuration, leading to a symmetric separatrix structure. In reality, Mercury's magnetic field is significantly asymmetric due to dipole tilt and interplanetary magnetic field (IMF) effects. Such asymmetries are expected to further disrupt adiabatic invariants and enhance particle losses. Recent studies (e.g., 10.1029/2024JA032987; 10.1029/2025GL116514) demonstrate that asymmetric configurations can strongly accelerate this process. I suspect this effect is particularly relevant for Mercury's radiation belt and encourage the authors to discuss it.

MESSENGER's magnetic field observations have shown that Mercury's magnetic dipole moment is closely aligned with the planet's rotation axis by <1° (Anderson et al., 2011). Dawn-dusk asymmetries at high altitudes in the magnetotail due to magnetospheric activities have been observed by MESSENGER (e.g., Sun et al., 2016), but such asymmetries have not been studied directly in the more dipolar closed field region. The dawn-dusk asymmetries in the "inner" dipolar closed field line region associated with the upstream interplanetary magnetic field (e.g., Kamaletdinov et al., 2024) or potential local crustal magnetic anomalies (e.g., Johnson et al., 2015), show potential influence on the bifurcation we are concerned about and worth mentioning in our main text.
We have added discussion (see <u>**Lines 419 to 420**</u>) to include radial transport driven by the asymmetric dawn-dusk magnetospheric field (Kamaletdinov et al., 2024, 2025), and localized loss induced by crustal magnetic anomalies (Johnson et al., 2015).

For planets with strong intrinsic magnetic fields (e.g., Earth, Jupiter, Saturn), radiation belt dynamics are largely governed by wave–particle resonant interactions, especially with whistler-mode waves. The authors' conclusions appear to suggest that such mechanisms are not significant at Mercury. This is an important point and should be explicitly stated and clarified in the manuscript.

The radiation belts of Earth, Jupiter, and Saturn, i.e., planets with stronger intrinsic magnetic fields, are largely governed by wave-particle interactions, particularly involving whistler-mode waves (Horne et al., 2005, 2008; de Soria-Santacruz et al., 2017, Roussos et al., 2021). Some of the particles in these radiation belts may also experience drift orbit bifurcation (e.g., within Earth's outer radiation belt, Shabansky, 1971). However, at Mercury, this non-adiabatic effect plays a more dominant role. This effect is particularly pronounced under strong solar wind driving and may compete with wave-particle interactions, as the rapid loss of electrons restricts the time available for them to interact with waves.

Nevertheless, our observations show that stably trapped electrons can reach higher energies than impulsive electrons (Figure 3d), which may point to the involvement of wave-induced acceleration. Under relatively weak solar wind conditions, electrons can be trapped for longer durations, allowing these magnetospheric waves to play a more significant role. Indeed, whistler waves, a prime candidate for such interactions, have recently been observed at Mercury (Ozaki et al., 2023). We have added these discussions in the revised manuscript in **Lines 380 to 383**.

Overall, this is a strong and timely contribution, and addressing the points above would further strengthen the paper.

Thank you!

References:

1. Anderson, Brian J., et al. "The global magnetic field of Mercury from MESSENGER orbital observations." *Science* 333.6051 (2011): 1859-1862.
2. Baker, Daniel N., et al. "Intense energetic electron flux enhancements in Mercury's magnetosphere: An integrated view with high-resolution observations from MESSENGER." *Journal of Geophysical Research: Space Physics* 121.3 (2016): 2171-2184.
3. Horne, Richard B., et al. "Wave acceleration of electrons in the Van Allen radiation belts." *Nature* 437.7056 (2005): 227-230.

4. Horne, Richard B., et al. "Gyro-resonant electron acceleration at Jupiter." *Nature Physics* 4.4 (2008): 301-304.
5. Johnson, Catherine L., et al. "Low-altitude magnetic field measurements by MESSENGER reveal Mercury's ancient crustal field." *Science* 348.6237 (2015): 892-895.
6. Kamaletdinov, S. R., et al. "Electron scattering due to asymmetric drift-orbit bifurcation: Geometric jumps of adiabatic invariant." *Journal of Geophysical Research: Space Physics* 129.11 (2024): e2024JA032987.
7. Kamaletdinov, S. R., et al. "Charged particle cross-field transport due to geometric jumps of adiabatic invariant." *Physical Review Letters* 135.6 (2025): 065202.
8. Ozaki, Mitsunori, et al. "Whistler-mode waves in Mercury's magnetosphere observed by BepiColombo/Mio." *Nature Astronomy* 7.11 (2023): 1309-1316.
9. Roussos, Elias, and Peter Kollmann. "The radiation belts of Jupiter and Saturn." *Magnetospheres in the solar system* (2021): 499-514.
10. Shabansky, V. P. "Some processes in the magnetosphere." *Space Science Reviews* 12.3 (1971): 299-418.
11. de Soria-Santacruz, M., et al. "Interactions between energetic electrons and realistic whistler mode waves in the Jovian magnetosphere." *Journal of Geophysical Research: Space Physics* 122.5 (2017): 5355-5364.
12. Sun, W. J., et al. "Spatial distribution of Mercury's flux ropes and reconnection fronts: MESSENGER observations." *Journal of Geophysical Research: Space Physics* 121.8 (2016): 7590-7607.

Reviewer #2 (Comments for the Author):

Zhao and Dewey et al. Dynamic electron radiation belt at Mercury controlled by solar wind driving. (2026)—> ZD26 hereafter.

ZD26 present observational evidence that Mercury's magnetosphere can magnetically confine and accelerate electrons over long dynamical timescales. The confirmation of long-lived trapped energetic electrons—i.e., radiation belts—at Mercury would constitute a landmark result, demonstrating that stable particle trapping can persist even in one of the most extreme and strongly driven magnetospheric environments in the Solar System. Such a finding would challenge the prevailing assumption that strong solar wind forcing, small system size, and weak magnetic fields inherently preclude sustained trapping, and would therefore require a revision of current theoretical frameworks in plasma physics and magnetospheric dynamics. More broadly, this result would suggest that key processes—such as adiabatic invariance, wave–particle interactions, and radial transport—remain effective, or reorganize in ways not yet fully understood, in regimes far from those typically considered. The implications extend beyond Mercury, with direct relevance for how radiation belts are modeled at Earth, other planets, and substellar magnetospheres.

I do not have specific reasons to question the validity of the observational evidence derived from the gamma-ray spectrometer (noting that this is not a dataset I have personally worked with). The proposed theoretical interpretation of the transition between trapped and untrapped orbits—based on enhanced solar wind perturbations near perihelion—is physically plausible and consistent with expectations. However, in its current form, the theoretical framework is not presented with sufficient clarity or justification to fully support the authors' conclusions. I therefore recommend major revision and provide below specific suggestions to improve the manuscript, particularly regarding the physical interpretation of the loss of confinement under strong driving conditions.

Comments:

1. The authors propose that the loss of confinement is primarily driven by drift-orbit bifurcation (last sentence p.9). However, the relevant sections (in particular the Appendix) are difficult to follow and appear to conflate distinct physical processes. Specifically, mechanisms associated with particle loss (e.g., bounce-unstable orbits arising from drift-orbit bifurcation) are not clearly distinguished from those responsible for acceleration and transport (e.g., radial advection and diffusion, which may also be influenced by drift-orbit

bifurcation). While all of these processes are in principle plausible, their respective roles are not sufficiently articulated or demonstrated for strong and weak driving conditions.

To improve clarity, we have reorganized **Section A.11 (Guiding Center Simulation)** by detailing each physical process in its own subsection: **A.11.1 Particle Initialization, A.11.2 Particle Tracing, and A.11.3 Particle Loss Evaluation**.

Specifically, particle loss is evaluated by checking for spatial intersection with the magnetopause (magnetopause shadowing), the planetary surface (direct precipitation), or the nightside stretched magnetic field. The latter is monitored using the particle's real-time κ parameter, where $\kappa < 3$ indicates strong curvature scattering that induces rapid pitch-angle scattering and subsequent precipitation onto the planetary surface.

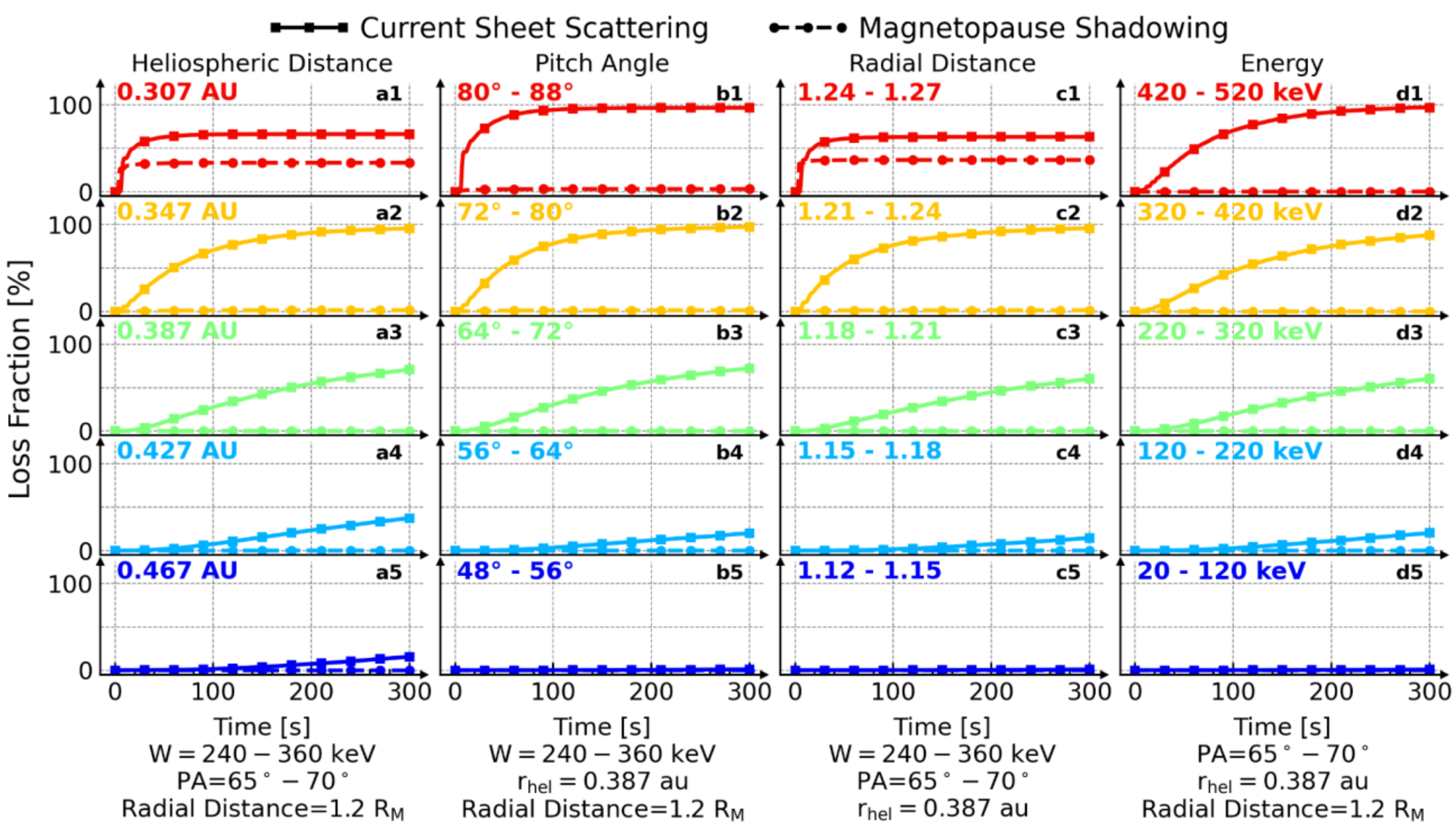

Figure R1. Contributions of the particle loss from current sheet scattering (solid lines with square markers) and magnetopause shadowing (dashed lines with circular markers). The four columns illustrate the sensitivity of particle loss to varying initial conditions: (a1–a5) heliocentric distance; (b1–b5) pitch angle; (c1–c5) radial distance; and (d1–d5) energy. For each column, the non-varying parameters are held constant, as indicated at the bottom of the respective panels. The layout and line colors of this figure follow those of Fig. 4e-4h in the main text. This figure has been added as Figure A5 in the revised manuscript.

In our simulations, most particle losses are attributed to either curvature scattering or magnetopause shadowing. Both mechanisms are promoted by the non-adiabatic radial

transport driven by drift orbit bifurcation. To distinguish their respective roles, we present their relative contributions to the total loss as a function of time in **Figure R1**. The results indicate that magnetopause shadowing only becomes comparable to curvature scattering during periods of high solar wind dynamic pressure (i.e., near perihelion), or when particle drift shells are further from the planet. Both scenarios correspond to a closer proximity between the drift orbits and the magnetopause, which confirm that the enhanced shadowing is physically sound.

We have incorporated this plot as **Figure A5**, along with the corresponding description and discussion, in **Section A.11.3 Particle Loss, Lines 964-969**.

Regarding the "bounce-unstable zone"**,** this geometric definition is utilized exclusively in our observational analysis. The "bounce-unstable zone" serves as a reference for determining a GRS/NS electron's trapping status based on its detected spatial position within the northward offset dipole geometry. We have expanded the description of the bounce-unstable zone in **Section A.10** to clarify its definition and application.

We would like to note that the simulations naturally incorporate the "the "bounce-unstable zone" since they are based on the KT17 magnetic field, in which the northward offset of the dipole center is included. This has also been noted in **Section A.10.**

In addition, the implementation of the theoretical framework lacks clarity. The authors appear to use the guiding-centre formalism of Brizard et al. (PoP) to compute particle trajectories, but the underlying magnetic field model is not specified. It is unclear whether the field is derived from the KT17 empirical model, and if so, how this model is incorporated into the guiding-centre Hamiltonian. In particular, the form of the vector potential, and its parametrization as a function of solar wind dynamic pressure, are not described.

We have clarified that the KT17 magnetic field model is utilized in our particle tracing as the underlying magnetic field in **section A.11 (Lines 941-943)**. In addition, we also clarified that the magnetic field vector, **B**, magnetic field direction, $\mathbf{b} \coloneqq \mathbf{B}/B$, correction term $\mathbf{B}^* \coloneqq \nabla \times \mathbf{A}^* = \mathbf{B} + p_\parallel/q\ (\nabla \times \mathbf{b})$, and associated spatial gradient/divergence/curl terms (e.g.,∇B) can all be directly computed from the KT17 model given a particle's parallel momentum in **section A.11 (Lines 937-940)**. Thus, all information required for the guiding-center tracing has been provided.

The KT17 model and its predecessor (KT14) modulate the influence of the magnetopause currents to account for the solar wind dynamic pressure, which is proportional

approximately to the inverse square of the heliocentric distance. Under enhanced solar wind dynamic pressure, the magnetopause currents intensify and move closer to the drift shells of radiation belt particles. Consequently, the particles undergo a more rapid transition in their bounce geometries due to the local field maxima near the dayside magnetic equator, which facilitates non-adiabatic radial transport and subsequent loss. **Figure R2** shows three examples of KT17 model magnetic field distributions and configurations under varying solar wind dynamic pressures.

We have added discussion of the solar wind dynamic pressure dependence of the KT17 magnetic field model in **Lines 822 to 830** and added **Figure R2** as **Figure A4** in **section A.9.**

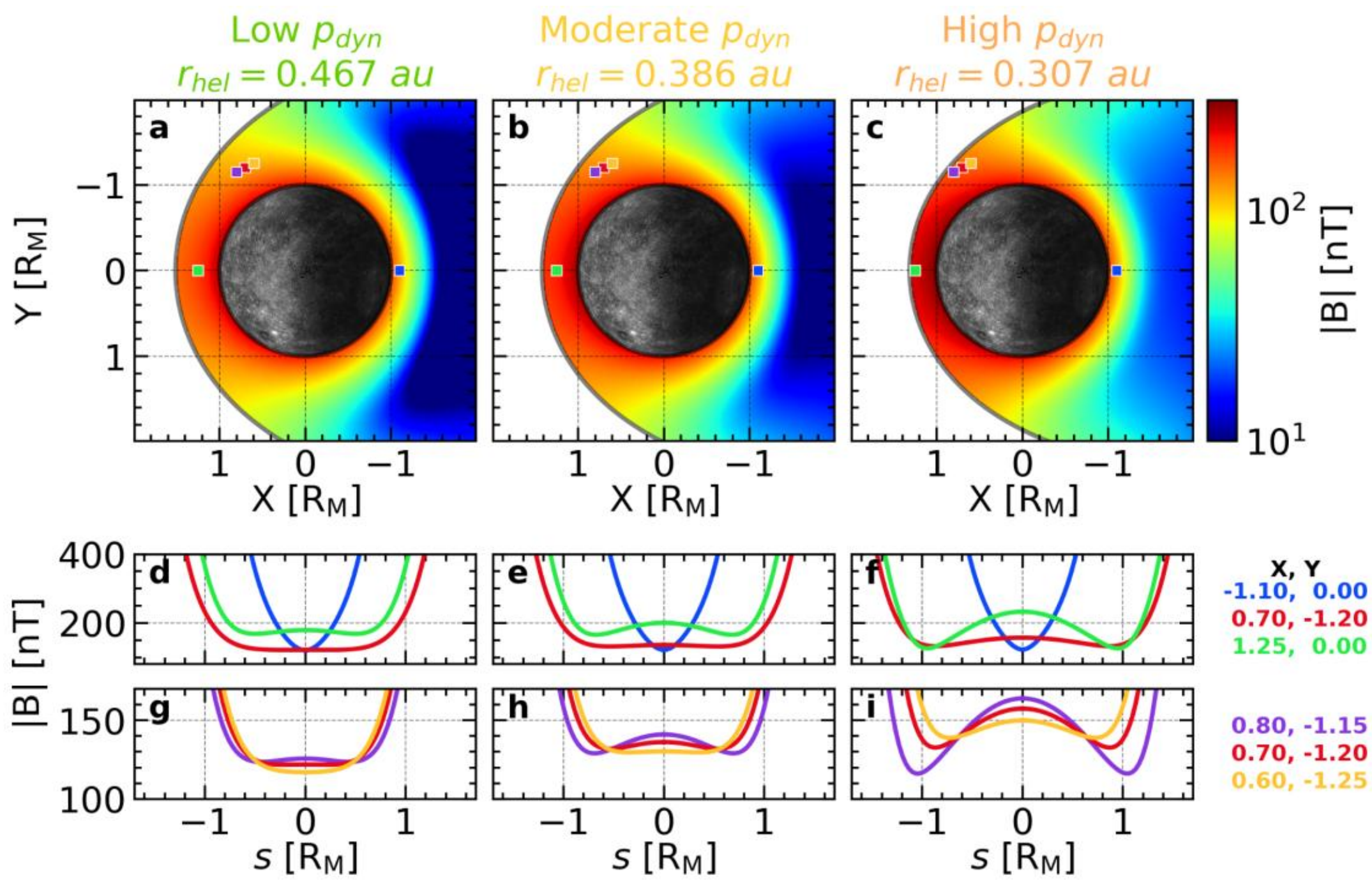

Figure R2. KT17 model magnetic field distributions and configurations under varying solar wind dynamic pressures (increasing from left to right). (**a-c**) Distributions of the magnetic field intensity in the magnetic equatorial plane. (**d-f**) Magnetic field intensity profiles along field lines on dayside (green), nightside (blue), and near the bifurcation (i.e., transition) point (red). (**g-i**) Magnetic field intensity for three magnetic field lines around the bifurcation point, which shows a significant change in the intensity profiles and bounce geometries.

This raises a more fundamental issue regarding the consistency between the different theoretical ingredients used in the manuscript. It is not evident how the KT17 magnetic field model connects to the drift-orbit Hamiltonian framework of Öztürk and Wolf (JGR, 2007), which is invoked to interpret radial transport (diffusive and/or convective). While

KT17 may be suitable to estimate the location of bounce-unstable regions (e.g., via Eq. A2), the dependence of the bifurcation parameters (e.g., the λ parameter) on the magnetic field configuration or on dynamical pressure is neither explained nor quantified.
More generally, although the Brizard formalism and the Öztürk and Wolf framework both originate from a Hamiltonian description, they are expressed in different variables. The manuscript does not make clear how these approaches are reconciled in practice. This lack of consistency, combined with the limited description of the numerical implementation, prevents a clear assessment of whether drift-orbit bifurcation alone can account for both the observed losses and the inferred acceleration of trapped electrons. Clarifying these points would significantly strengthen the physical interpretation and improve confidence in the proposed mechanism.

We appreciate the interest in these technical details. When we prepared our submission, we hesitated on whether to include the full theoretical methodology and decided that the toy-model was a balance between simplicity and detail. However, we are glad to include a full description of our theoretical foundation. Below provides a summary. A detailed theoretical work has been provided in the **supporting document**.

In the full description of the bifurcation model (Öztürk and Wolf, 2007; Ukhorskiy et al., 2011), the phase space coordinates, which is the displacement along the magnetic field line, s, and guiding center's parallel velocity, $v_\parallel$, are closely aligned with the Brizard's formalism, in which we can define s by $\frac{\mathrm{d}s}{\mathrm{d}t} = \mathbf{b} \cdot \frac{\mathrm{d}\mathbf{X}}{\mathrm{d}t}$ and parallel momentum $p_\parallel$ by $\gamma m v_\parallel$.

In the revised supporting information, we have replaced the simplified bifurcation theory starting from a toy model ($H = \frac{p^2}{2} - \lambda \frac{q^2}{2} + \frac{q^4}{4}$) with its full description with step-by-step derivation. This full description more clearly shows how the radial transport efficiency is positively correlated with how rapidly the bouncing geometry transitions from the conventional, inter-hemispheric bounce path to the bifurcated one (i.e., Equation 30 in the supporting information).

This quantitative relationship explains why the loss rate increases as particles' drift velocity increases (e.g., higher kinetic energy) and/or their drift orbits get closer to the magnetopause current (e.g., high p_{dyn} or outer drift shell), which is the primary cause of the dayside equatorial magnetic field intensification. The more rapid transition between bounce geometry at higher p_{dyn} is also shown in the response above in the last paragraph.

The toy model appears to be redundant after the inclusion of the full theory, and it has been introduced by several previous literatures (e.g., Cary et al., 1986, Öztürk and Wolf,

2007 and Ukhorskiy et al., 2011). Thus, we have removed the toy model in our revised supporting information.

2. The authors understate the significance of their results in the Introduction. The potential discovery of radiation belts at Mercury would not only provide a distinct laboratory for studying space weather processes relevant to Earth, but also probe regimes that are largely inaccessible in the terrestrial magnetosphere. Mercury's magnetosphere operates under far more extreme conditions and may, in some respects, be viewed as an analogue of Earth's response to rare, highly disturbed Carrington-like events.

More importantly, the implications extend beyond the Earth–Mercury comparison. The existence of radiation belts at Mercury would directly inform outstanding questions on particle acceleration and transport across a wide range of planetary and substellar magnetospheres (DOI: 10.1126/sciadv.aea4945), which operate in regimes significantly different from Earth (e.g., strong vs weak co-rotation, synchrotron losses, stellar-wind-versus rotation-driven systems). As noted by the authors (footnote 2), the drift period of energetic particles at Mercury is comparable to the injection timescale, implying a system that is persistently driven far from local thermodynamical equilibrium. This is a qualitatively different regime from that typically assumed for Earth's radiation belts. In this context, Mercury could serve as a critical testbed for the validity of current quasilinear wave–particle acceleration models, including both local interactions and radial transport (DOI:10.3847/1538-4365/acff6a). These models generally rely on assumptions of weak departures from equilibrium and diffusive saturation of instabilities associated with loss and acceleration processes—assumptions that are unlikely to hold under Mercury-like conditions. This broader significance should be more clearly articulated, particularly for an astronomy and astrophysics readership that is often more focused on fundamental plasma processes in such environments than on space weather specifically.

We thank the reviewer for this suggestion. We agree that emphasizing the broader implications strengthens the manuscript and helps place its findings in a wider context. We have added brief text to the Abstract (**Lines 63 to 65**), Main (**Lines 79 to 80**), and Conclusions (**Lines 439 to 447**) sections to highlight why Mercury's radiation belt is important for the Earth-Mercury comparison (and beyond) and how it can inform radiation belt physics more broadly.

3. As far as drift-orbit bifurcation is concerned, the authors may wish to consider the following recent study, which accounts for more realistic asymmetric potentials in the Hamiltonian and provides an estimate of the radial transport advection timescale that can be compared to their observations: DOI: https://doi.org/10.1103/nsg2-scpg.

The asymmetries associated with the upstream interplanetary magnetic field (e.g., Kamaletdinov et al., 2024) or local magnetic anomaly (e.g., Johnson et al., 2015), show potential influence on the bifurcation we are concerned about and worth mentioning in our main text.

We have added discussion (see **lines 419 to 420**) to include enhanced radial transport driven by the asymmetric dawn-dusk magnetospheric field (Kamaletdinov et al., 2024, 2025), and localized loss induced by crustal magnetic anomalies (Johnson et al., 2015).

4. Regarding Section A.9, the authors state that the KT17 model shows strong agreement with observed magnetic field data, citing a root mean square residual of 20–30 nT. However, this metric would be more informative if placed in the context of varying solar wind driving conditions. Given the large variability of Mercury's magnetic field strength across different regions and levels of external forcing, an absolute residual expressed in nT is difficult to interpret in terms of model accuracy. In particular, it is unclear whether a 20–30 nT discrepancy represents a small or significant deviation under weak versus strong driving conditions.

Providing a normalized measure of the residual (e.g., relative to the local magnetic field strength), or stratifying the error as a function of dynamical pressure, would help clarify the level of agreement and its relevance for the physical processes discussed. It should also be noted that, in the case of drift-orbit bifurcation, it is not only the magnetic field magnitude that matters, but also its gradients, and it is not clear to what extent this can or has been quantified for KT17.

We agree that the 20-30 nT root mean square (RMS) residual statistics are too simplistic. We have reorganized the model's residuals provided by Korth (2022) in NASA's Planetary Data System. In Figure R3, we evaluate the absolute and relative residual between the observation and model under different solar wind dynamic pressure $\left(p_{\mathrm{dyn}}\right)$ conditions (as indicated by magnetopause subsolar standoff distance, r_{ss}) with 4 types of metrics: RMS (red squares), upper-quartile, median, and lower-quartile (black vertical error bars). Figure R3 shows that both absolute and relative residuals tend to increase under stronger solar wind forcing. In the most significant case, the RMS absolute residual reaches ~43 nT with a relative residual ~ 22% when $r_{\mathrm{ss}} < 1.3\ \mathrm{R_M}$. However, the 75th and 50th percentiles of the residuals are ~18% and 12%, respectively.

Among the r_{ss} bins, the upper quartiles of the residuals are less or comparable to the RMS values, i.e., in about 75% of cases, the actual model-observation discrepancy is smaller than what the RMS residual implies. This indicates that the RMS is likely inflated by a small percentage of large deviations, which could be attributed to a few scenarios.

For example, the diamagnetism from the cusp (e.g., Dewey et al., 2025), which corresponds to open field lines and thus would not affect the particle tracing simulation results; or the field-aligned currents (e.g., Anderson et al., 2014).

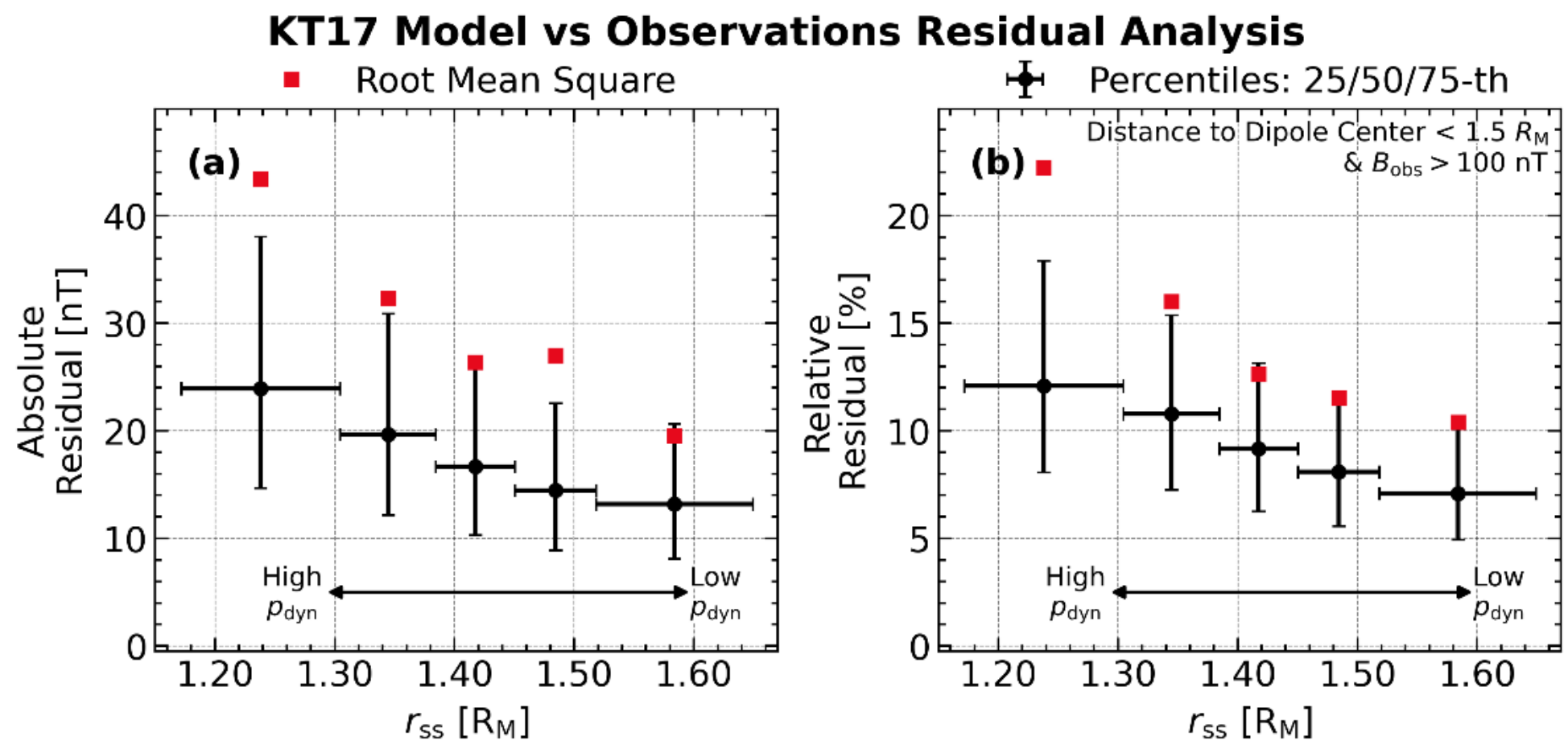

Figure R3. Residual analysis of the KT17 model compared to observations. (a) Absolute residuals ($\delta B = |\mathbf{B}_{\mathrm{KT17}} - \mathbf{B}_{\mathrm{obs}}|$). (**b**) Relative residuals ($\delta B / B_{\mathrm{obs}}$). The red squares and vertical black error bars represent the root-mean-square and percentiles (25th, 50th, and 75th) of these residuals, respectively. Horizontal error bars denote the width of each subsolar magnetopause distance (r_{ss}) bin. Each bin contains data from over 700 orbits. When calculating the relative residuals, the observational data are restricted to radial distances within 1.5 Mercury radii ($\mathrm{R_M}$) and measured magnetic field strengths (B_{obs}) greater than 100 nT. These criteria correspond to the inner dipolar region where radiation belt particles drift.

Regarding the magnetic field gradient, we have addressed this in response to your first comment (please refer to **Figure R2** and the accompanying text).

In conclusion, in the radiation belt region, the model's results align with the physical expectations. The residuals relative to the local magnetic field are around 10% indicating that the model captures the large-scale physics. Thus, the model is considered acceptable.

We have expanded A9 (**Lines 822-830**) and added **Figure A4** (**Figure R2** in this reply) to discuss and visualize this dependence of the KT17 model on solar wind dynamic pressure and the development of a local maximum in the equatorial magnetic field and transition from conventional to bifurcated bounce paths. The residual analysis (**Figure R3**) has been included in the supporting information.

Reviewer #2 (Remarks on code availability):

At present, no README or equivalent documentation appears to be provided for the Python codes, which makes it difficult to assess their intended usage, dependencies, and relation to the results shown in the manuscript. More importantly, the code used to process the gamma-ray spectrometer data—the crucial dataset underlying the identification of radiation belts at Mercury—is written in IDL. I was not able to evaluate this part of the workflow directly, in part because no documentation is provided and in part because IDL requires proprietary licensed software. As a result, the most critical step of the analysis is not readily reproducible from the materials currently described.

All codes developed to analyze the data are now publicly available on Zenodo (Dewey, 2026, Doi:10.5281/zenodo.19663490; Zhao, 2026, Doi:10.5281/zenodo.19475783). In those Zenodo depositories, we have provided a complete project description, a user manual, a demo, and example outputs.

References:

1. Anderson, Brian J., Catherine L. Johnson, and Haje Korth. "A magnetic disturbance index for Mercury's magnetic field derived from MESSENGER Magnetometer data." *Geochemistry, Geophysics, Geosystems* 14.9 (2013): 3875-3886.
2. Anderson, Brian J., et al. "Steady-state field-aligned currents at Mercury." *Geophysical Research Letters* 41.21 (2014): 7444-7452.
3. Dewey, Ryan M., et al. "Interpreting energy-latitude dispersions in Mercury's northern magnetospheric cusp with MESSENGER." *Journal of Geophysical Research: Space Physics* 130.8 (2025): e2025JA034278.
4. Johnson, Catherine L., et al. "Low-altitude magnetic field measurements by MESSENGER reveal Mercury’s ancient crustal field." *Science* 348.6237 (2015): 892-895.
5. Kamaletdinov, S. R., et al. "Electron scattering due to asymmetric drift-orbit bifurcation: Geometric jumps of adiabatic invariant." *Journal of Geophysical Research: Space Physics* 129.11 (2024): e2024JA032987.
6. Kamaletdinov, S. R., et al. "Charged particle cross-field transport due to geometric jumps of adiabatic invariant." *Physical Review Letters* 135.6 (2025): 065202.
7. Korth, Haje, et al. "A dynamic model of Mercury's magnetospheric magnetic field." *Geophysical Research Letters* 44.20 (2017): 10-147.
8. Korth, H. "MESSENGER Magnetometer KT17 Model Mercury Solar Orbital (MSO) Residual Data." *NASA Planetary Data System* (2022): 24. Access URL: https://pds-ppi.igpp.ucla.edu/collection/urn:nasa:pds:mess-mag-kt17-model-residuals:data-sci-mso

Reply to reviewers

We thank both reviewers for their suggestions. Below is a point-by-point response to all comments from this round. A revised manuscript has been included in the submitted documents.

We also note that the manuscript has been revised according to the editorial requirements, including: (1) merging the original footnote into the main text; (2) adjusting the figure size and their font sizes; (3) revising the figure captions; and (4) rearranging the section order. Additionally, we have replaced the symbol for the mean signal-to-noise ratio from μ to $\overline{\mathrm{SNR}}$, as μ is already used to denote the magnetic moment. These editorial changes are highlighted in blue in the revised manuscript. All figures and scientific content remain unchanged.

Reviewer #1 (Comments for the Author):

Authors have addressed all my comments and i'm ready to recommend this paper for publication

Thank you for your suggestions and comments!

Reviewer #2 (Comments for the Author):

The authors have carefully addressed my previous concerns and suggestions, and the manuscript is now in excellent shape. However, the exchange with the other reviewer appears to have introduced a minor issue that I would ask the authors to revise.

The statement that radiation belt dynamics at Jupiter and Saturn are “largely governed by wave–particle resonant interactions, especially with whistler-mode waves” appears to be an overgeneralization from the terrestrial case and is not supported by the review cited by the authors (Roussos & Kollmann). At Earth, wave–particle interactions (e.g., chorus and hiss) indeed play a dominant role for electrons in the ~10 to hundreds of keV range and are central to both acceleration and loss processes. However, this framework does not directly extend to the giant planets.

The cited review emphasizes that Jupiter and Saturn operate in a fundamentally different regime, where magnetospheric dynamics are largely internally driven (due to rapid planetary rotation and strong plasma sources from moons), rather than solar-wind driven. As a result, several processes play a primary role in shaping the radiation belts, including: (1) radial transport (both diffusive and convective) and associated adiabatic acceleration,

(2) interchange instability, which drives rapid inward transport of <MeV particles,
(3) corotation and large-scale electric fields, and
(4) strong coupling to moons, rings, and neutral material, which act as both sources and sinks.

Wave–particle interactions are discussed in the cited work, but not as the dominant controlling mechanism. For Saturn, local acceleration by whistler-mode chorus is described as inefficient, with electron populations primarily controlled by radial transport and loss processes. For Jupiter, chorus waves may contribute to energizing freshly injected particles but become inefficient once distributions reach steady state, while radial transport remains a key energy source.

Furthermore, for ions and for large portions of the electron populations at both planets, dominant processes include CRAND (especially for Saturn's MeV protons), radial diffusion, interchange injections, charge exchange, and absorption by moons and rings. These processes differ significantly from the standard Earth radiation belt paradigm and often dominate over wave-driven diffusion.

Therefore, the statement in its current form is misleading and should be revised. A more accurate formulation would be that wave–particle interactions are one of several contributing processes, while radial transport, corotation-driven dynamics, and moon–magnetosphere coupling play a central—often dominant—role at Jupiter and Saturn, depending on particle species and energy.

We thank the reviewer for the detailed comments and explanations of this point. We agree with the point brought by the reviewer.

The relevant sentence in the "Conclusion" section has been modified according to the reviewer's suggestion with some small wording to better fit the flow. The current sentence is

"At Earth, wave–particle interactions play a central role in these processes, whereas at Jupiter and Saturn, Cosmic Ray Albedo Neutron Decay (CRAND), radial transport, interchange instabilities, corotation-driven dynamics, and strong coupling to moons and rings are often dominant, with wave–particle interactions acting as one of several contributing mechanisms [4, 26, 27, 38]"

We have retained the discussion in the "Results" section of whistler-mode wave-particle interactions, both as a potential mechanism for accelerating Mercury's radiation belt electrons, and as a common, though not necessarily dominant, contributor to electron

acceleration in the radiation belts of Earth and the giant planets. We have added further references to complement ref. [4] for the reader's convenience.

"For example, wave-particle interactions, particularly with whistler modes [26, 27], are reported to accelerate the stably trapped radiation belt electrons up to megavolt energies in the more intense planetary fields of Earth, Jupiter and Saturn [4, 28-30], and may be a candidate for acceleration of these electrons at Mercury."

References

[4] Roussos, E. & Kollmann, P. The radiation belts of Jupiter and Saturn. *In Magnetospheres in the Solar System*, **Ch. 32**, 499–514 (American Geophysical Union, Hoboken, NJ, 2021).

[26] Horne, R. B. et al. Wave acceleration of electrons in the Van Allen radiation belts. *Nature* **437**, 227–230 (2005).

[27] Horne, R. B. et al. Gyro-resonant electron acceleration at Jupiter. Nat. Phys. **4**, 301–304 (2008)

[28] Woodfield, E. E., Horne, R. B., Glauert, S. A., Menietti, J. D. & Shprits, Y. The origin of Jupiter's outer radiation belt. J. Geophys. Res. Space Phys. 119, 3490–3502 (2014).

[29] de Soria-Santacruz, M. et al. Interactions between energetic electrons and realistic whistler mode waves in the Jovian magnetosphere. *J. Geophys. Res. Space Phys*. **122**, 5355–5364 (2017).

[30] Woodfield, E. et al. Rapid electron acceleration in low-density regions of Saturn's radiation belt by whistler-mode chorus waves. *Geophys. Res. Lett.* **46**, 7191–7198 (2019).

[38] Osmane, A., Kilpua, E., George, H., Allanson, O. & Kalliokoski, M. Radial transport in the Earth's radiation belts: Linear, quasi-linear, and higher-order processes. *Astrophys. J. Suppl. Ser*. **269**, 44 (2023).

Reviewer #2 (Remarks on code availability):

README files are now provided and steps by steps details for installation are clearly explained.

Thank you!
